# Analysis of genetic profiling, pathomics signature, and prognostic features of primary lymphoepithelioma‐like carcinoma of the renal pelvis

**DOI:** 10.1002/1878-0261.13307

**Published:** 2022-09-02

**Authors:** Bo Fan, Yuanbin Huang, Hongshuo Zhang, Tingyu Chen, Shenghua Tao, Xiaogang Wang, Shuang Wen, Honglong Wang, Zhe Lin, Tianqing Liu, Hongxian Zhang, Tao He, Xiancheng Li

**Affiliations:** ^1^ Department of Urology Second Affiliated Hospital of Dalian Medical University China; ^2^ Department of Biochemistry, Institute of Glycobiology Dalian Medical University China; ^3^ Department of Clinical Medicine Dalian Medical University China; ^4^ Department of Pathology Dalian Friendship Hospital China; ^5^ Ethics Committee Second Affiliated Hospital of Dalian Medical University China

**Keywords:** genetic characteristics, immunohistochemical profile, lymphoepithelioma‐like carcinoma, population‐based study, propensity score matching, whole‐genome sequencing

## Abstract

The genetic features of primary lymphoepithelioma‐like carcinoma (LELC) of the upper urinary tract have not been systematically explored. In this study, tumor mutation profiling was performed using whole‐genome sequencing in two patients with LELC of the renal pelvis. Novel candidate variants relevant to known disease genes were selected using rare‐variant burden analysis. Subsequently, a population‐based study was performed using the Surveillance, Epidemiology, and End Results (SEER), PubMed, MEDLINE, Embase, and Scopus databases to explore clinical features and prognostic risk factors. Immunohistochemical analysis revealed seven positive cytokeratin‐associated markers in tumor cells and five positive lymphocyte‐associated markers in and around the tumor area. Sub‐sequently, we identified *KDM6A* as the susceptibility gene and *LEPR* as the driver gene by Sanger sequencing in case 2 of LELC of the renal pelvis. Three mutation sites of the existing targeted drugs were screened: *CA9*, a therapeutic target for zonisamide; *ARVCF*, a therapeutic target for bupropion; and *PLOD3*, a therapeutic target for vitamin C. In a population‐based study, patients with primary LELC of the upper urinary tract had clinical outcomes similar to those of patients with primary upper urinary tract urothelial carcinoma (UUT‐UC) before and after propensity score matching at 1 : 5. Focal subtype was an independent prognostic factor for the overall survival of patients with LELC of the upper urinary tract. The carcinogenesis of primary LELC may be due to different genetic variations, including single‐nucleotide variants, insertion and deletions, structural variations, and repeat regions, which may provide the basis for clinical diagnosis and treatment. The prognosis of LELC in the upper urinary tract is similar to that of UUT‐UC. We suggest that the focal subtype can serve as a prognostic factor for LELC of the upper urinary tract; however, further studies are required to confirm this.

## Graphical abstract

Here, the genetic, pathological and prognostic features of primary lymphoepithelioma‐like carcinoma (LELC) of the renal pelvis are explored. Mutations in the driver gene *LEPR* and susceptibility gene *KDM6A* might be associated with LELC of the renal pelvis. The prognosis of LELC of the upper urinary tract is similar to that of UUT‐UC.

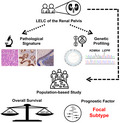

**Abbreviations**
AA: amino acid
Alt: alternative
BAM: binary alignment map
BWA: Burrows‐Wheeler Aligner
CCF: cancer cell fraction
CD: cluster of differentiation
CDS: coding sequence
Chr: chromosome
CIs: confidence intervals
CK: cytokeratin
CNV: copy number variation
COSMIC: the Catalogue of Somatic Mutations in Cancer Database
CT: computed tomography
CTX: chromosomal translocations
DAB: 3,3′‐diaminobenzidine
DEL: deletion
FDA: Food and Drug Administration
FFPE: formalin‐fixed and paraffin‐embedded
FPG: fusion partner gene
GATA3: GATA binding protein 3
HCV: hepatitis C virus
HRs: hazard ratios
ICD: International Classification of Diseases
INDELs: insertions and deletions
LELC: lymphoepithelioma‐like carcinoma
N/A: not applicable
ncRNA: noncoding ribonucleic acid
NGS: second‐generation sequencing
NMF: non‐negative matrix factorization
p63: protein‐63
PCR: polymerase chain reaction
PharmGKB: the Pharmacogenomics Knowledge Base Database
PSM: propensity score matching
qPCR: quantitative polymerase chain reaction
Ref: reference
RN: radical nephrectomy
RNU: radical nephroureterectomy
SEER: Surveillance, Epidemiology, and End Results
SMG: significantly mutated gene
SNPs: single‐nucleotide polymorphisms
SNVs: single‐nucleotide variants
SVs: structural variations
TNM: tumor, node and metastasis
Trf: transformation
TS : TV: transformation/transmutation ratio
UC: urothelial bladder carcinoma
UCSC hg: the University of California, Santa Cruz human reference genome
UTR: untranslated region
UUT‐UC: upper urinary tract urothelial carcinoma
WGS: whole‐genome sequencing

## 1 Introduction

Lymphoepithelioma, characterized by syncytial nests of malignant epithelial cells with a prominent reactive lymphoid infiltrate, is an undifferentiated epithelial tumor primarily found in the nasopharynx and is especially common in young Asian populations [1, 2, 3]. Tumors with histological features similar to those of other organ systems are lymphoepithelioma‐like carcinomas (LELC). LELC was later identified in carcinomas of the breast [4], esophagus [5], stomach [6], and lungs [7]. LELC of the renal pelvis is a rare histological subtype of aggressive upper urinary tract carcinoma first reported in 1998 [8].

In the current study, two cases of LELC of the primary renal pelvis are presented. A comprehensive genetic analysis of the two cases was performed using whole‐genome sequencing (WGS). Given the lack of data on the prognosis and characteristics of LELC in the upper urinary tract, LELC and upper urinary tract urothelial carcinoma (UUT‐UC) cases were added from the Surveillance, Epidemiology, and End Results (SEER), PubMed, MEDLINE, Cochrane, Web of Science, Embase, and Scopus databases. From reviewing related literature and public databases, combined with data from our two cases, clinicopathologic features, therapeutic strategies, and prognosis were evaluated.

## 2 Materials and methods

### 2.1 Tissue samples

The relevant clinical characteristics of case 1 (a 61‐year‐old man, who pathologically diagnosed with lymphoepithelioma‐like carcinoma of the renal pelvis) were obtained from the First Affiliated Hospital of Dalian Medical University, whereas the related clinical characteristics of case 2 (a 76‐year‐old woman, who pathologically diagnosed with lymphoepithelioma‐like carcinoma of the renal pelvis) were obtained from the Second Affiliated Hospital of Dalian Medical University. This project was approved by the Ethics Committee of the First Affiliated Hospital of Dalian Medical University (No. LCKY2015‐08) and the Second Affiliated Hospital of Dalian Medical University (No. DYEY‐2022‐018). The study methodologies conformed to the standards set by the Declaration of Helsinki. Written informed consents to participate in the study were obtained from the patients for use of their samples. Patient consent for publication by using samples.

### 2.2 Immunohistochemical analysis

Formalin‐fixed tissues were paraffin‐embedded and cut into 5‐μm sections. After dewaxing in xylene and hydration in ethanol, sections were placed in sodium citrate buffer for antigen repair and heated at 95 °C for 20 min. The sections were incubated with 0.5% hydrogen peroxide for 20 min to block endogenous peroxidase activity and blocked with goat serum for 1 h, followed by overnight incubation with primary antibodies at 4 °C. Antibody information is shown in Table S1. Sections were incubated with an avidin‐biotin kit according to the manufacturer's instructions. After developing the chromogen 3,3′‐Diaminobenzidine (DAB) for 5 min at near 24 °C and counterstaining with hematoxylin for 30 s, the sections were observed using a microscope (Leica, Wetzlar, Germany).

### 2.3 Whole‐genome sequencing

#### 2.3.1 DNA extraction

Genomic DNA was extracted from two formalin‐fixed and paraffin‐embedded (FFPE) LELC tissues and two matched normal renal cortical tissues using the GeneRead DNA FFPE Kit (Qiagen, Hilden, Germany), following the manufacturer's instructions. The quantity and purity of genomic DNA were assessed using 1% agarose gel electrophoresis to analyze DNA degradation and impurities, and using a Qubit® 2.0 Fluorometer (Invitrogen, Carlsbad, CA, USA) to quantify DNA concentration.

#### 2.3.2 Library preparation and sequencing

Whole‐genome sequencing libraries were captured using the Agilent SureSelect Human All Exon Kit (Agilent Technologies, Santa Clara, CA, USA), according to the manufacturer's recommendations. Genomic DNA was randomly fragmented into 350 bp fragments using a Covaris instrument (Covaris, Woburn, MA, USA). The products were purified using an AMPure XP system (Beckman Coulter, Beverly, MA, USA). Quality control was performed using a Qubit® 2.0 Fluorometer (Invitrogen), Agilent Bioanalyzer 2100, and a quantitative polymerase chain reaction (qPCR) approach to quantify library concentration and evaluate library quality. Sequencing libraries were sequenced on an Illumina HiSeq platform (Illumina, San Diego, CA, USA) using the Novogene sequencing facility (Novogene, Beijing, China). Sanger sequencing of the susceptibility and driver genes was performed by Sangon Biotech Co., Ltd. (Sangone Biotech, Shanghai, China).

#### 2.3.3 Quality control

The filtration of raw data containing adapter reads, undetected nucleotides, and low‐quality nucleotides is essential for obtaining clean reads for quality analysis. After removing the following reads: adapter reads, reads with the proportion of unconfirmed base information greater than 10%, and paired reads with the proportion of low‐quality (Phred quality < 5) bases greater than 50%, subsequent analysis was based on the obtained high‐quality clean reads.

#### 2.3.4 Bioinformatics analysis

Sequencing reads were aligned to the University of California, Santa Cruz human reference genome (UCSC hg19) using Burrows‐Wheeler Aligner (bwa, 0.1.22) software with binary alignment map (bam) file generation [9, 10]. picard (http://broadinstitute.github.io/picard/) and sambamba software (v0.4.7) [11] were used for duplicate read marking, repetition processing, and bam file sorting. The sequence coverage and depth were calculated by sequence alignment.

#### 2.3.5 WGS data processing and mutation analysis

The samtools software (1.0) was used to test for single‐nucleotide polymorphisms (SNPs) and insertions and deletions (INDELs) [12]. INDELs, structural variations (SVs), and single‐nucleotide variants (SNVs) were detected using the mutect (1.1.4) [13], strelka (v1.0.13) [14], crest (v0.0.1) [15], and control‐freec (v6.7) softwares [16]. Finally, the mutation results were annotated using annovar (2013 Aug 23) software [17]. We classified 96 mutation types (4 × 6 × 4) according to the type of base at 1 bp upstream and downstream of somatic SNV and the six possible mutations at this site. The mutation signature analysis is based on the frequency of 96 mutation types in tumor samples by non‐negative matrix factorization (NMF) to factorize somatic SNV into several different mutation signatures, and the factorized mutation signatures were compared with the known mutation signatures in the Catalogue of Somatic Mutations in Cancer (COSMIC) database [18] to explain the mutation process of two samples.

#### 2.3.6 Analysis of potential driver mutations, susceptibility genes

Sample mutations were compared with known driver mutations in the Bert Vogelstein [19], significantly mutated gene (SMG) [20], Comprehensive [21], and Cancer Gene Census (http://cancer/sanger.ac.uk/cancergenome/projects/census) databases to identify potential driver genes for LELC in the renal pelvis. SIFT, Polyphen‐2, and Mutation‐Taster scores were used to assess whether the mutations were pathogenic. Moreover, after detecting germline mutations (SNVs and INDELs) in the normal tissue of matched patients using samtools software, potential susceptibility genes could be identified by detecting germline mutations and comparing them with those in the Cancer Gene Census database and two susceptibility gene databases [22, 23] using in‐house software. Based on the results of structural variation, SV events with breakpoints in the gene region were identified as possible gene fusions.

#### 2.3.7 Analysis of tumor purity, tumor ploidy, and clonal structure and the screening for resistant mutations

We used absolute [24] and pyclone [25] for purity, ploidy, and cancer cell fraction (CCF) analyses of both samples. The absolute software calculates the purity and ploidy of tumor samples based on copy number and somatic mutation frequency. To analyze tumor evolution, pyclone software was used to analyze tumor clonal structure by using somatic mutation frequency of the samples combined with tumor purity, copy number, and other information to calculate CCF. Cluster analysis was performed on the tumor cells to determine the clonal structure of the tumor samples. Based on the detection of somatic mutations in tumor samples, the detected mutation sites were compared with the NovoDR drug‐resistant gene databases [26] to screen for possible cancer drug‐resistant mutations.

### 2.4 Population‐based study

#### 2.4.1 Data resource and study population

The clinicopathologic features and survival data of UUT‐UC and LELC of upper urinary tract patients were obtained from the SEER database, which contains official clinicopathologic and follow‐up reports from 18 population‐based tumor registries that mainly embody the U.S. patient population [27]. The following inclusion criteria for UUT‐UC patients were used: (a) transitional cell carcinoma, NOS [International Classification of Diseases (ICD)‐0‐38120/3]; (b) transitional cell carcinoma, spindle cell carcinoma (ICD‐0‐38122/3); (c) papillary transitional cell carcinoma (ICD‐0‐38130/3); and (d) transitional cell carcinoma, micropapillary (ICD‐0‐38131/3). Cancer diagnosis was determined based on positive histological outcomes for the first time. Patients whose histological and survival data were lost were excluded. Based on these criteria, a final cohort of 18 183 UUT‐UC patients was included in the present analysis. Additionally, the PubMed, Medline, Cochrane, Web of Science, Embase, and Scopus databases were searched to identify relevant studies examining LELC of the upper urinary tract from database inception until March 2022 (*n* = 39) [8, 28, 29, 30, 31, 32, 33, 34, 35, 36, 37, 38, 39, 40, 41, 42, 43, 44, 45, 46, 47, 48, 49, 50, 51, 52, 53, 54, 55]. The main search terms included: ‘lymphoepithelioma‐like carcinoma’, ‘ureter’, ‘renal pelvis’, ‘upper tract urothelial carcinoma’, ‘upper urinary tract urothelial carcinoma’, ‘prognosis’, ‘survival’, and ‘case report’. The SEER database was used (lymphoepithelial carcinoma, ICD‐0‐38082/3; *n* = 5). Finally, combined with the two cases in this study, 46 cases with LELC of the upper urinary tract were included. The flow diagram of the LELC cases is shown in Fig. S1.

#### 2.4.2 Clinicopathological characteristics

Baseline patient characteristics and outcome data included sex, age, race, tumor location, tumor focality, tumor side, pathological classification, surgery type, lymphadenectomy, and application of chemotherapy and radiation therapy. Eligible patients who were not clearly stated were classified using version 7 of the tumor, node, metastasis (TNM) classification system of malignant tumors, according to the full‐text description. The main endpoint was overall survival, which was defined as the time from the initial diagnosis of cancer to death from any cause or the last follow‐up, according to the literature and the SEER database. Patients who were still alive at the last follow‐up were censored.

### 2.5 Statistical analyses

Clinicopathological characteristics were assessed to determine the significant differences between upper urinary tract LELC and UUT‐UC. Fisher's exact probability and Pearson's chi‐square tests were used for categorical and continuous variables, respectively. Hazard ratios (HRs) and 95% confidence intervals (CIs) for different survival‐related variables were calculated using the Cox proportional hazards model. The two histological types were compared using Kaplan–Meier plots and log‐rank tests. spss version 13.0 (IBM Corp., Armonk, NY, USA) was used for all statistical analyses. To eliminate potential confounding factors in the clinicopathological baseline characteristics, propensity score matching (PSM) was conducted using r software version 3.6.0 (http://www.R‐project.org/). One LELC patient was matched with five UUT‐UC patients by using the predetermined clinicopathological factors described above. Statistical significance was defined as a two‐sided *P*‐value < 0.05.

## 3 Results

### 3.1 Case characteristics

**Fig. 1:**
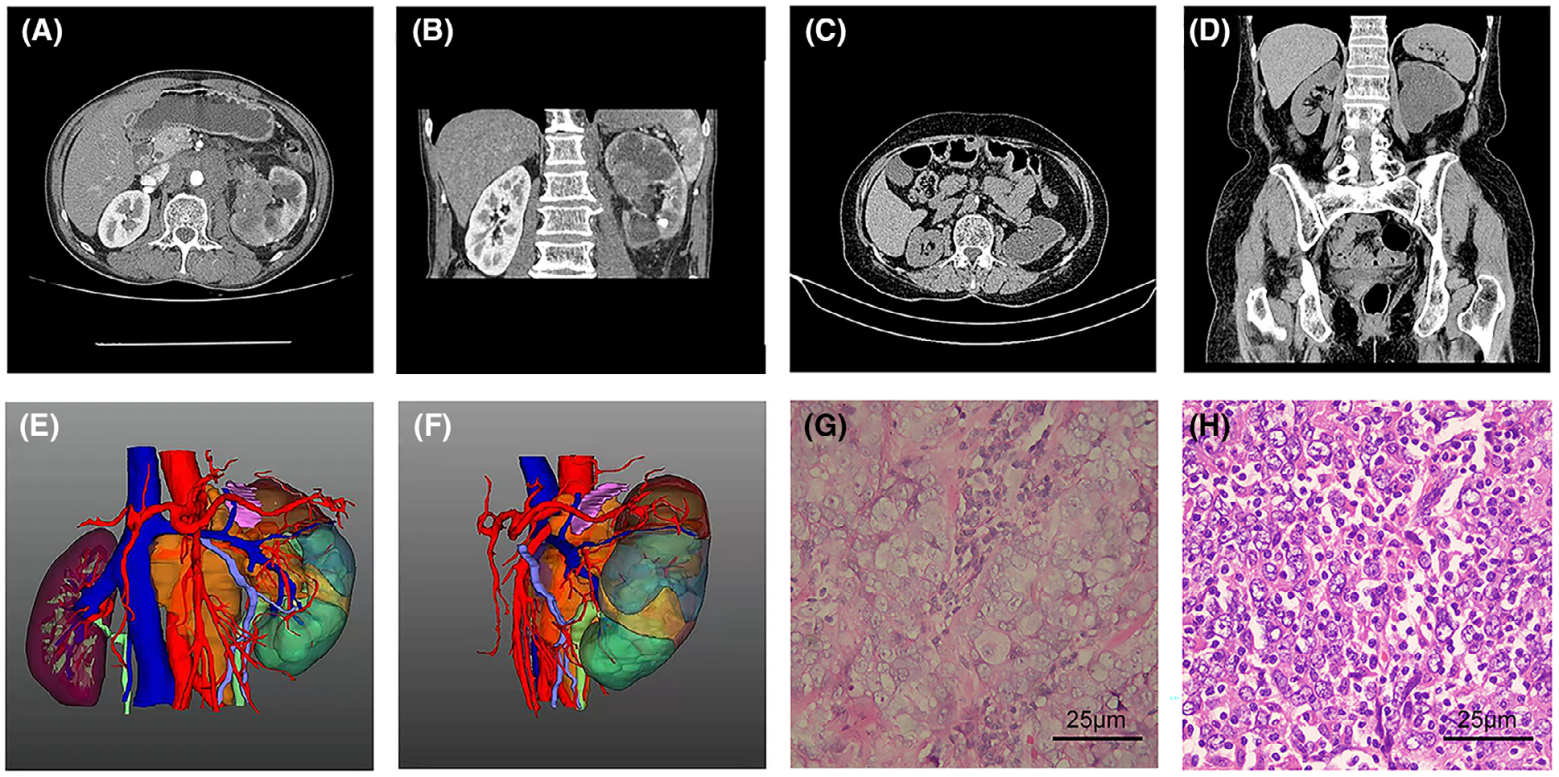
Radiological and pathological findings of the primary lymphoepithelioma‐like carcinoma of the renal pelvis. (A, B) Computed tomography (CT) images of the urinary system of case 1 patient from coronal and sagittal views showing a tumor in the left renal pelvis and uronephrosis in the left renal pelvis and multiple lymph node metastases in the left renal hilus and the peritoneum. (C, D) Abdominal CT of case 2 patient from coronal and sagittal views revealing a stricture between the renal pelvis and ureter with no function of the left kidney. Coronal (E) and axial (F) views of reconstructed three‐dimensional images clearly show the tissue mass and surrounding anatomical structure. Hematoxylin and eosin staining of cancer tissue samples was obtained from case 1 (G) and 2 (H) (*n* = 2, scale bar: 25 μm).

Case 1 was a 61‐year‐old man who presented with swelling and pain on the left side of his waist for 2 months. Enhanced computed tomography (CT) images of the urinary system revealed a tumor in the left renal pelvis, tumor invasion into the upper section of the left ureter, swelling and uronephrosis in the left renal pelvis, and multiple lymph node metastases in the left renal hilus and peritoneum (Fig. 1A). Ultrasound examination of the urinary system showed severe uronephrosis in the left kidney, with a width of 35 mm and a weak echo in the area (53 mm × 38 mm). Pulmonary CT revealed scattered nodules in both lungs (Fig. 1B), with the largest nodule being approximately 1.07 cm in size, suggesting metastatic disease in the lungs. Moreover, CT showed enlargement of the mediastinal lymph nodes, the largest of which was 0.72 cm in diameter. Urine cytology showed one atypical specimen and two positive specimens in three consecutive urinations. No abnormalities were observed in the bladder during the cystoscopy. The patient was clinically diagnosed with left renal pelvic carcinoma with multiple metastases in the lungs and underwent left radical nephroureterectomy (RNU) under continuous epidural anesthesia. The patient was pathologically diagnosed with a primary lymphoepithelioma‐like carcinoma of the left renal pelvis. The patient did not receive any adjuvant chemotherapy or radiotherapy postoperatively and eventually died of the disease 9 months later.

Case 2 involved a 76‐year‐old woman with left hydronephrosis and ureteral stricture that persisted for 3 months. Ultrasonography revealed enlargement of the left kidney with an anechoic renal cortical cyst and severe dissociation of the left renal collecting system with a width of 36 mm, suggesting severe left uronephrosis and a renal pelvic ureteral transitional lesion. Abdominal computed tomography revealed a stricture between the renal pelvis and the ureter (Fig. 1C,D). Dynamic renal imaging revealed no abnormalities in her left kidney. Urine cytology revealed one atypical and two negative tumor cell findings in three consecutive urinations. No abnormalities were observed in the bladder during the cystoscopy. Our clinical results suggested a diagnosis of congenital stenosis, given the nonfunctioning left kidney, and the patient subsequently underwent laparoscopic left nephrectomy under general anesthesia. During surgery, we observed a cauliflower‐like neoplasm in the renal pelvis, and frozen sections sampled at the time of surgery revealed high‐grade urothelial carcinoma. RNU with excision of the bladder cuff was laparoscopically performed. Pathological examination of the surgical specimen confirmed a preoperative diagnosis of lymphoepithelial carcinoma. The patient did not receive any adjuvant chemotherapy or radiotherapy postoperatively and eventually died of the disease 15 months later. Three‐dimensional images were reconstructed according to the CT results, which showed the tissue mass and the surrounding anatomical structure (Fig. 1E,F).

### 3.2 Pathological features study

#### 3.2.1 Histopathological presentation

Hematoxylin–eosin staining was performed on pathological sections of tumor tissues from the two patients. In case 1, the tumor cells were arranged in lamellar nests with high atypia, a high nucleolus ratio, prominent nucleoli, and eosinophilic nuclei. Lymphocytes infiltrated the stroma and were scattered around the nests of tumor cells (Fig. 1G). In case 2, the tumor cells showed patchy growth and marked atypia with vacuolated nuclei and small nucleoli. Marked infiltration of lymphocytes was observed between the tumor cells (Fig. 1H).

#### 3.2.2 Immunohistochemical profile

**Fig. 2:**
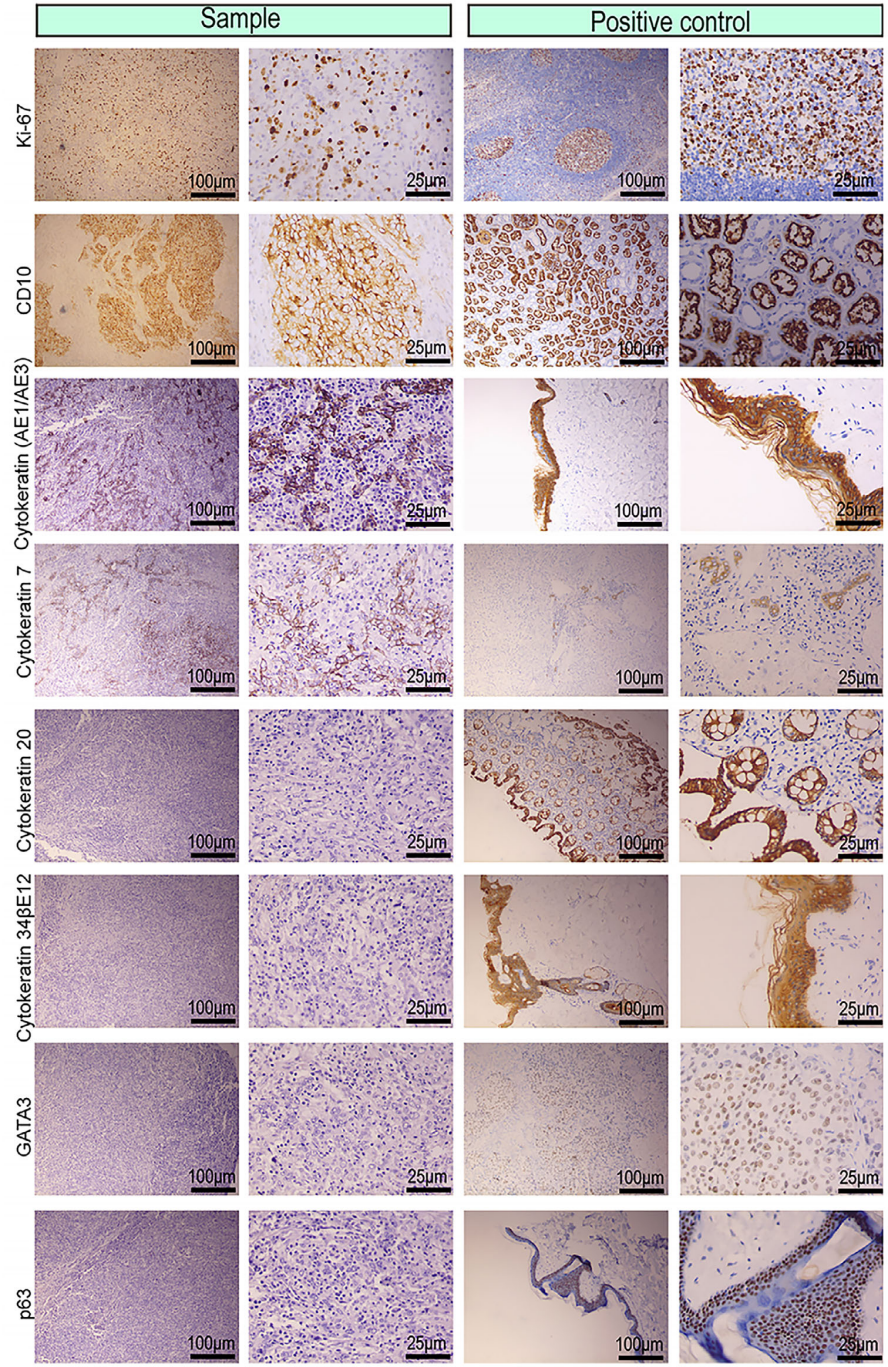
Photomicrographic immunohistochemical images of the tumor cells from the primary lymphoepithelioma‐like carcinoma of the renal pelvis. Representative immunohistochemical images for Ki‐67, cluster of differentiation (CD) 10, Cytokeratin AE1/AE3, Cytokeratin 7, Cytokeratin 20, Cytokeratin 34βE12, GATA binding protein 3 (GATA3), and protein‐63 (p63), which were positive in tumor cells, indicating the markers of lymphoepithelioma‐like carcinoma (*n* = 2, scale bar: 100 μm, 25 μm).

**Fig. 3:**
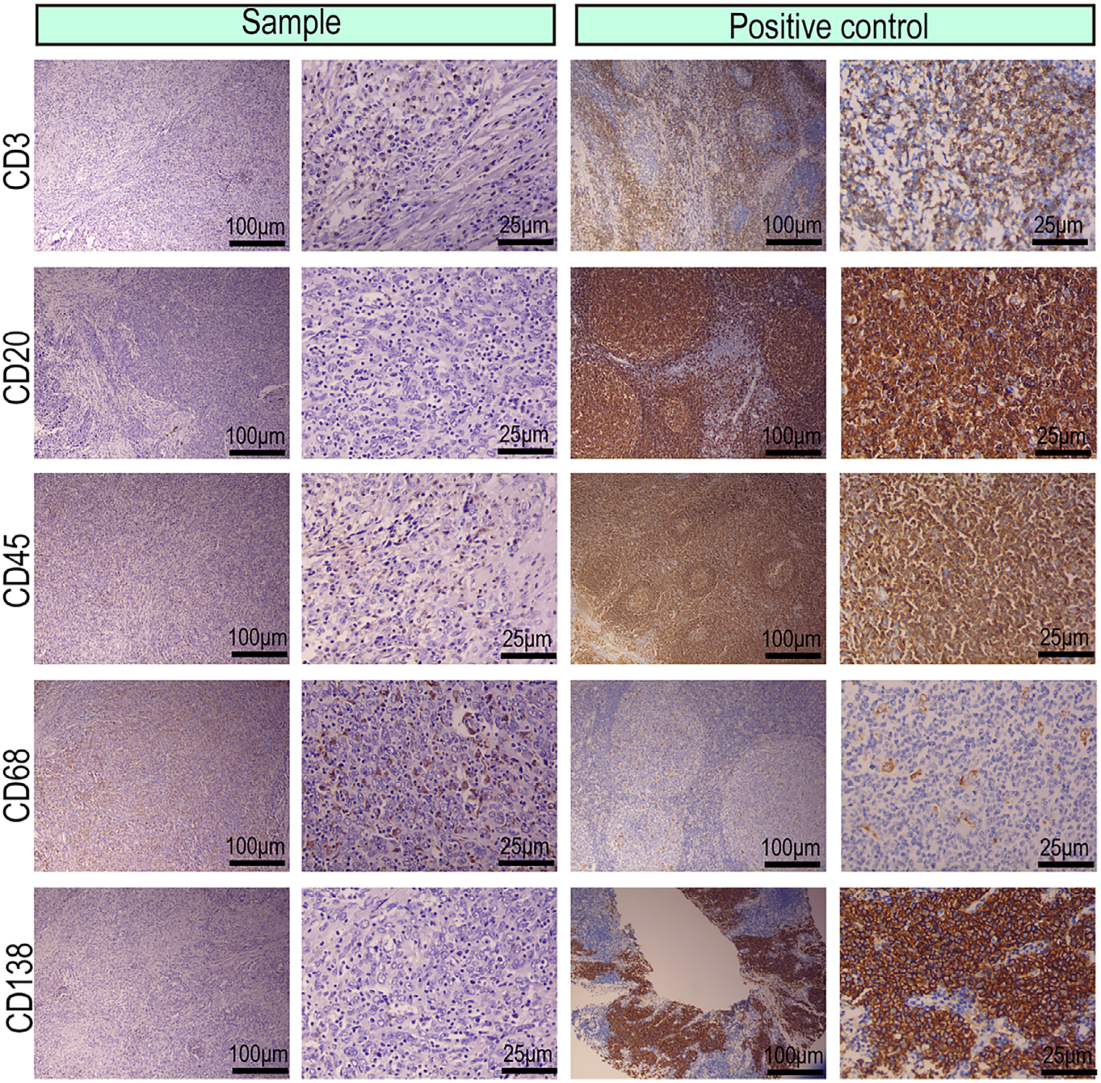
Photomicrographic immunohistochemical images of the stromal lymphocytes from the primary lymphoepithelioma‐like carcinoma of the renal pelvis. Representative immunohistochemical images for cluster of differentiation (CD) 3, CD20, CD45, CD68, and CD138, which were negative in tumor cells, indicating the markers for tumor stromal lymphocytes (*n* = 2, scale bar: 100 μm, 25 μm).

Immunohistochemical analysis revealed histopathological manifestations of a primary lymphoepithelioma‐like carcinoma of the renal pelvis. The Ki‐67 staining was strongly positive. The tumor cells were positive for cluster of differentiation (CD) 10, cytokeratin (CK) AE1/AE3, cytokeratin 7, cytokeratin 20, cytokeratin 34βE12, GATA binding protein 3 (GATA3), and protein‐63 (p63), which are markers of lymphoepithelioma‐like carcinomas (Fig. 2). Additionally, for the differential diagnosis of lymphoma, tumor cells were detected with negative or no dominant staining of CD3, which is a T‐lymphocyte marker; CD20, which is a B‐lymphocyte marker; and CD45, which is a lymphocyte marker. For the differential diagnosis of plasmacytoma, tumor cells were detected with negative or non‐dominant staining for CD138, a plasma cell marker. For the differential diagnosis of various mononuclear histiocytic‐derived tumors or malignant fibrous histiocytoma, tumor cells were detected with negative or no dominant staining of CD68, a macrophage marker (Fig. 3).

### 3.3 Whole‐genome sequencing study

#### 3.3.1 WGS identification of SNPs and INDELs

**Fig. 4:**
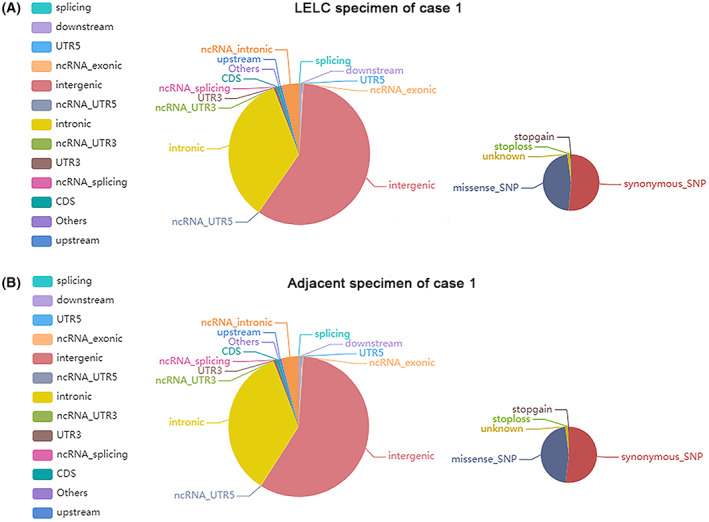
Distribution of single‐nucleotide variants in case 1. The images show the number of single‐nucleotide variants in different regions of the genome (left) and the coding regions (right) after sequencing the cancer (A) and adjacent (B) specimens. CDS, coding sequence; UTR, untranslated region. All the experiments were repeated thrice independently.

**Fig. 5:**
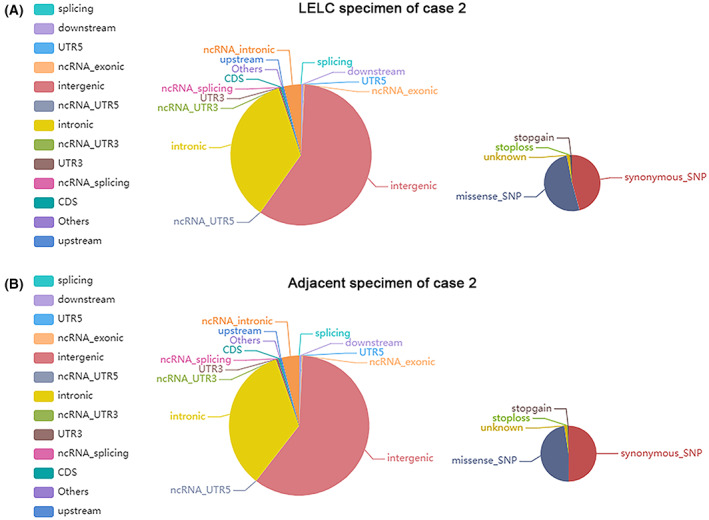
Distribution of single‐nucleotide variants in case 2. The images show the number of single‐nucleotide variants in different regions of the genome (left) and the coding regions (right) after sequencing the cancer (A) and adjacent (B) specimens. CDS, coding sequence; UTR, untranslated region. All the experiments were repeated thrice independently.

**Fig. 6:**
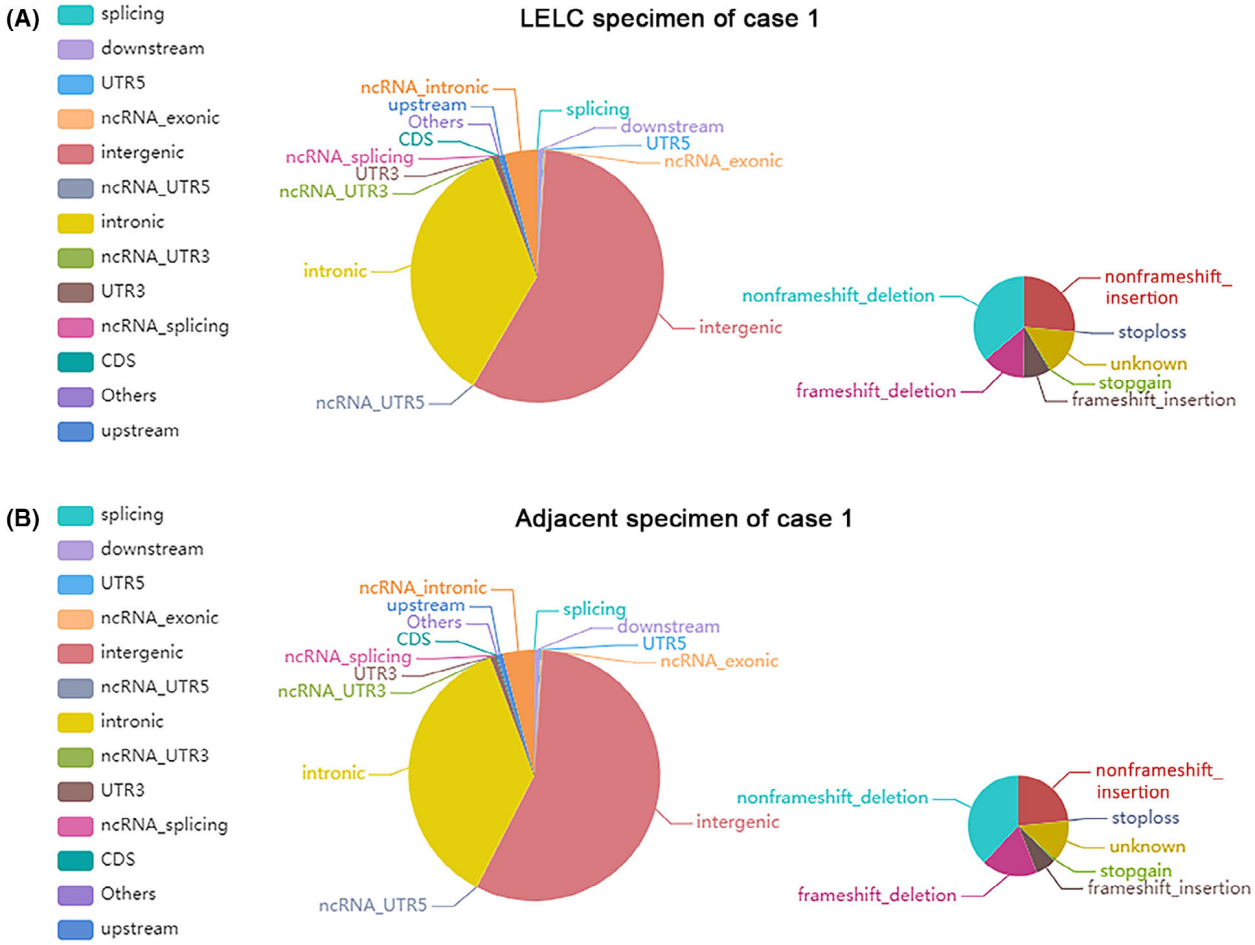
Distribution of insertions and deletions in case 1. The images show the number of insertions and deletions in different regions of the genome (left) and the coding regions (right) after sequencing the cancer (A) and adjacent (B) specimens. CDS, coding sequence; UTR, untranslated region. All the experiments were repeated thrice independently.

**Fig. 7:**
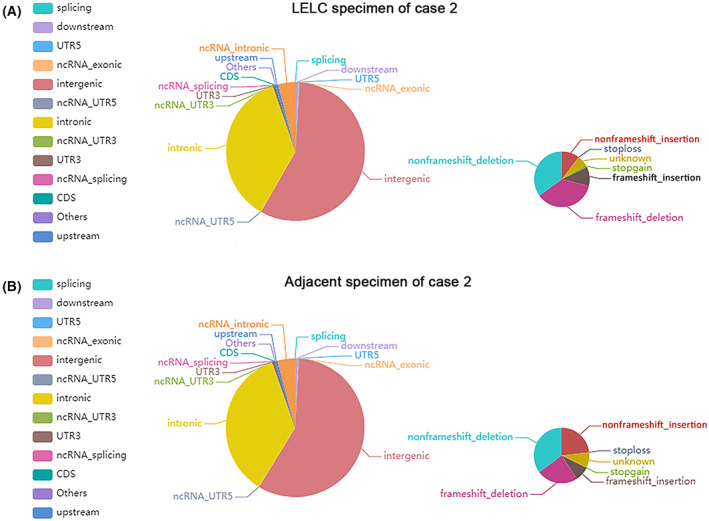
Distribution of insertions and deletions in case 2. The images show the number of insertions and deletions in different regions of the genome (left) and the coding regions (right) after sequencing the cancer (A) and adjacent (B) specimens. CDS, coding sequence; UTR, untranslated region. All the experiments were repeated thrice independently.

To investigate the genetic basis of LELC, WGS was performed with a mean proportion of Q30 > 80% and a mean error rate < 0.1% in the primary LELC specimens and matched normal tissues from the renal cortex of the two cases, respectively. All variants were annotated using the annovar software. We sequenced 656 400 250 (case 1) and 267 512 090 (case 2) read pairs in the primary tumor and 635 040 964 (case 1) and 397 049 253 (case 2) read pairs in normal tissue specimens. A total of 3 268 638 SNPs in case 1 and 1 754 424 in case 2 were identified in the LELC specimen: 2 821 722 and 2 708 028 in the adjacent normal specimen of cases 1 and 2, respectively. The transformation/transmutation ratio (TS : TV) was employed for the exactness of the SNP dataset, which was approximately 2.2 in the whole genome and approximately 3.2 in the coding region. An individual has approximately 350 000 INDELs in its genome. A total of 523 417 in case 1 and 267 005 in case 2 were detected in the LELC specimen; 414 198 in case 1 and 412 892 in case 2 were found in the adjacent normal specimens. Most of the identified SNPs and INDELs were located in intergenic and intronic regions (Tables S2–S5). The number of SNPs and INDELs in different regions of the genome and the number of SNPs and INDELs in different types of coding regions are shown in Figs 4, 5 and Figs 6, 7, respectively.

#### 3.3.2 Analysis of somatic SNVs and INDELs

Somatic mutations occurring in normal cells are the basis for our study of driver genes, fusion genes, and tumor resistance. The outcomes of somatic mutations in the two cases are shown in Fig. S2. mutect was utilized to detect somatic SNV sites, and 10 110 and 2682 SNVs were identified in cases 1 and 2, respectively, mainly distributed in the intergenic, intronic, and noncoding ribonucleic acid (ncRNA) intronic regions (Table S6). For INDELs, we applied strelka to identify somatic INDEL information, including detected 63 INDELs in case 1 and 1206 INDELs in case 2, predominantly located in intronic and intergenic regions (Table S7).

#### 3.3.3 Analysis of structural variations and repeat regions

**Fig. 8:**
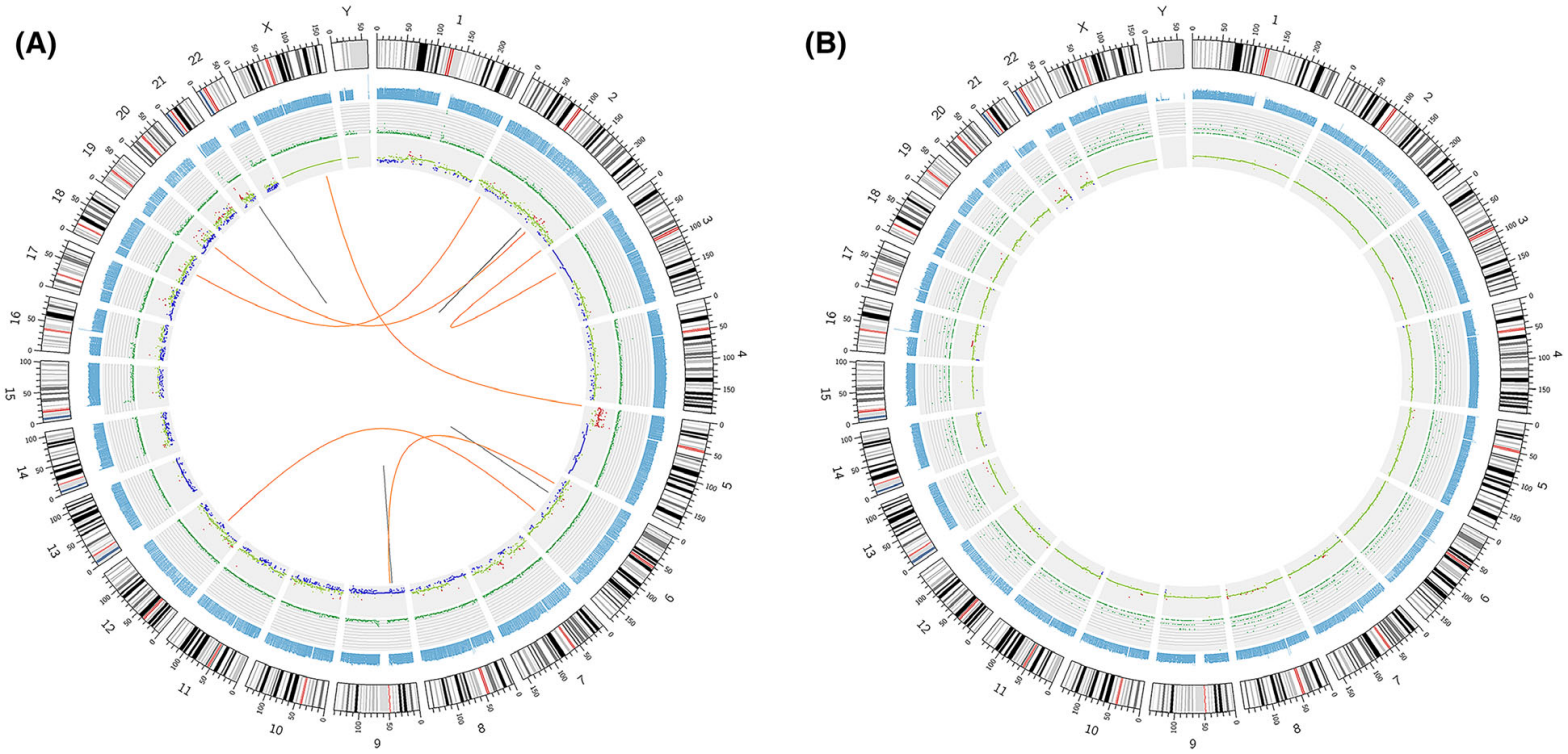
Genomic variation circos display in the primary lymphoepithelioma‐like carcinoma of the renal pelvis. (A) Genomic variation circus for case 1. (B) Genomic variation circus for case 2. The five‐layer structure from the outside to the inside represents the sequencing coverage map, the density of karyotype stripe, single‐nucleotide variant, insertions and deletion, copy number variation, and the structural variation results, respectively. All the experiments were repeated thrice independently.

**Fig. 9:**
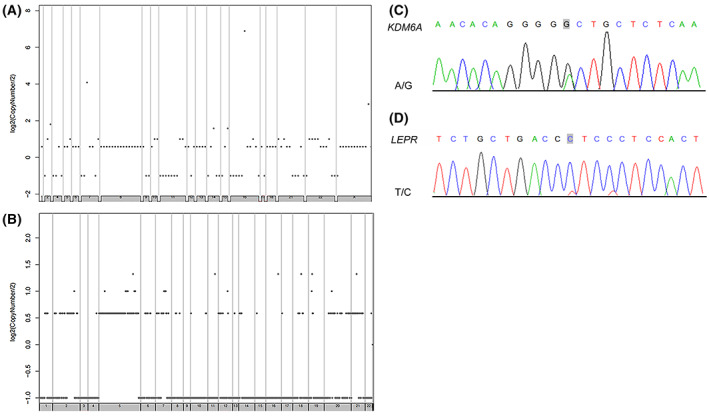
Chromosome plots and the Sanger sequencing results. (A) Chromosome plots showing copy number variation (CNV) results in case 1. (B) Chromosome plots showing CNV results in case 2. (C) Results of nucleotide Sanger sequencing analysis of *KDM6A*. Sanger sequencing electropherograms of the *KDM6A* mutant at position chromosome (Chr) X: 44928980 A>G. (D) Results of nucleotide Sanger sequencing analysis of *LEPR*. Sanger sequencing electropherograms of the *LEPR* mutant at position Chr1: 66096026T>C. All the experiments were repeated thrice independently.

Structural variation which comprises deletion, insertion, duplication, copy number variants, inversion, and translocation is shown in Table S8. We counted the number of SVs of interchromosomal translocations (CTX) and deletions (DEL). Copy number variation (CNV) results were classified into two types: deletion and duplication. In case 1, we identified seven CTXs, four DELs, and 776 CNVs. Nonetheless, no CTX or DEL were detected in case 2, and 119 CNVs were identified. Finally, we used the Circos tool to show somatic cell variation in the two LELC samples (Fig. 8A,B). We then used chromosome plots to show the CNV results (Fig. 9A,B). Detailed information regarding the tandem repeat regions identified in the primary LELC of the renal pelvic tissue is presented in Table S9.

#### 3.3.4 Analysis of susceptibility genes and genes with driver mutations

Susceptibility gene mutation is defined as a genetic alteration that increases an individual's susceptibility or predisposition to a certain disease or disorder. Susceptibility genes can encode proteins involved in inherited diseases or can confer disease susceptibility in appropriate environments [56, 57]. The samtools software was used to detect germline mutations (SNPs and INDELs). The results were filtered using the database mentioned in Section 2 to screen for possible cancer susceptibility genes. The results are presented in Table S10. The driver mutation is a term used to describe changes in the DNA sequence of genes that cause cells to become cancer cells and grow and spread in the body. Driver gene mutation provides tumors with a selective growth advantage and has an important effect on the proliferation and diffusion of tumors [58, 59]. We compared genes with somatic variations with known driver genes and screened out known driver genes in primary tumor samples. The results of the driver gene analysis are presented in Table S11. In addition, polymerase chain reaction (PCR) amplification was used for secondary confirmation and Sanger sequencing was performed on the susceptibility and driver genes that might contain mutant bases. Analysis of germline DNA showed a G mutant base in the sequence of *KDM6A* (Fig. 9C), and *LEPR* had a T mutant base (Fig. 9D) in case 2. *KDM6A* and *LEPR* were validated as the susceptibility and driver genes of LELC, respectively.

#### 3.3.5 Analysis of tumor purity, ploidy, and clonal structure

As tumor tissues may contain impurities, the purity (the proportion of tumor cells to total cells) and ploidy (the average copy number of the sample) of tumor samples were calculated to ensure the quality of analysis. absolute software was employed for the computer with a purity of 0.5 in both cases, and the ploidy was 4.58 in case 1 and 2.03 in case 2. Moreover, the proportion of tumor DNA in cases 1 and 2 was 70% and 50%, respectively. To explore the evolutionary process of tumors, their clonal structure was analyzed. The cancer cell fraction, which is the critical basis for pyclone to study the cluster structure, refers to the proportion of tumor cells carrying a certain mutation in all tumor cells. The closer the CCF value is to 1, the more likely it is that this mutation is an early one common to all tumor cells, namely major clonal mutation; a smaller CCF value indicates that only a subset of tumor cells have this mutation, namely subclonal mutation. The top five major mutant clones were *CNTNAP3*, *NFXL1*, *KIAA1147*, *CD200R*1, and *NBPF9*. Single‐sample clonal structure analysis of the two cases was performed to study intratumoral heterogeneity; the results are shown in Fig. S3.

#### 3.3.6 Analysis of targeted drug prediction

After comparing the identified somatic mutations and the Novo Drug database, including the Pharmacogenomics Knowledge Base Database (PharmGKB), My Cancer Genome, and the Food and Drug Administration (FDA) databases, we screened three mutation sites of the existing targeted drugs *CA9*, *PLOD3*, and *ARVCF* in two cases, for which detailed information is available in Table 1. There were four drugs (zonisamide, hydroflumethiazide, hydrochlorothiazide, and benzthiazide) for *CA9*, two drugs (bupropion and risperidone) for *ARVCF* and vitamin C for *PLOD3*.

**Table 1:** Prediction of targeted drugs for high‐frequency mutation genes in two cases. AA, amino acid; Chr, chromosome; N/A, not applicable.

Gene symbol	Entriz ID	Chr	Start position	End position	Variant classification	AA change	Drug name	Drug type	Interaction type	Source
*PLOD3*	8985	7	100853896	100853896	Missense mutation	*PLOD3*:NM_001084:exon13:c.C1417G:p.R473G	Vitamin C	Small molecule	N/A	DrugBank
*PLOD3*	8985	7	100853896	100853896	Missense mutation	*PLOD3*:NM_001084:exon13:c.C1417G:p.R473G	Succinic acid	Small molecule	N/A	DrugBank
*CA9*	768	9	35679968	35679968	Missense mutation	*CA9*:NM_001216:exon8:c.G1183T:p.D395Y	Benzthiazide	Small molecule	Inhibitor	DrugBank
*CA9*	768	9	35679968	35679968	Missense mutation	*CA9*:NM_001216:exon8:c.G1183T:p.D395Y	Hydroflumethiazide	Small molecule	Inhibitor	DrugBank
*CA9*	768	9	35679968	35679968	Missense mutation	*CA9*:NM_001216:exon8:c.G1183T:p.D395Y	Zonisamide	Small molecule	Inhibitor	DrugBank
*CA9*	768	9	35679968	35679968	Missense mutation	*CA9*:NM_001216:exon8:c.G1183T:p.D395Y	Hydrochlorothiazide	Small molecule	Inhibitor	DrugBank
*ARVCF*	421	22	19969503	19969503	Missense mutation	*ARVCF*:NM_001670:exon4:c.T322C:p.S108P	Bupropion	Efficacy	N/A	Pharm GKB
*ARVCF*	421	22	19969503	19969503	Missense mutation	*ARVCF*:NM_001670:exon4:c.T322C:p.S108P	Risperidone	Efficacy	N/A	Pharm GKB
*LEPR*	3953	1	66096026	66096026	Missense mutation	*LEPR*:NM_001198687:exon19:c.C2815T:p.L939F	Simvastatin	Efficacy	N/A	Pharm GKB
*LEPR*	3953	1	66096026	66096026	Missense mutation	*LEPR*:NM_001198687:exon19:c.C2815T:p.L939F	Simvastatin	Efficacy	N/A	Pharm GKB
*LEPR*	3953	1	66096026	66096026	Missense mutation	*LEPR*:NM_001198687:exon19:c.C2815T:p.L939F	Simvastatin	Efficacy	N/A	Pharm GKB

### 3.4 Population‐based study

#### 3.4.1 Comparison between LELC of the upper urinary tract and UUT‐UC

##### 3.4.1.1 Patient clinical and demographic characteristics

Until March 2022, 46 patients with LELC of the upper urinary tract identified in the public database and 18 183 patients with UUT‐UC extracted from the SEER database were included in our study. Table 2 shows the detailed clinicopathological characteristics of the two patient cohorts. There were significant differences in age (≥ 72 years, 52.2% vs. 48.2%; *P* < 0.001) and the proportion of sex (male, 58.7% vs. 42.2%; *P* < 0.001) between LELC patients and UUT‐UC patients, as well as statistical differences across the three races (*P* < 0.001). For gross and histological features, the LELC group, which was relative to the UUT‐UC group, tended to be unifocal (*P* = 0.002) and had significant differences in tumor sides (*P* < 0.001). Compared with the UUT‐UC group, the LELC group had a higher stage (T_2_–T_4_, 78.3% vs. 14.9%; *P* < 0.001), higher lymph node involvement (positive lymph node status, 26.1% vs. 4.6%; *P* < 0.001), and lower incidence of distant metastasis (M_1_, 0.0% vs. 3.6%; *P* < 0.001). Regarding the treatment modality, patients with LELC of the upper urinary tract were more likely to undergo surgery, especially radical nephroureterectomy and nephrectomy (100.0% vs. 56.6%; *P* < 0.001), whereas no significant differences were detected in chemotherapy or radiation. The overall clinical and pathological data of the two groups after propensity score matching are shown in Table 3. After performing 1 : 5 PSM for baseline factors and treatments to eliminate selection bias, 34 patients with LELC and 166 with UUT‐UC were included. The results showed significant differences in tumor side (*P* = 0.035) and pathological stage (*P* = 0.016).

**Table 2:** Baseline characteristics of patients with lymphoepithelioma‐like carcinoma of the upper urinary tract and upper urinary tract urothelial carcinoma. The bold values were applied to highlight *P*‐values, which had statistically significance (i.e., *P* < 0.05).

Characteristics	LELC group (*n* = 46)	UUT‐UC group (*n* = 18 183)	Total	*P*‐value
Gender				
Male	27	7678	7705	**<0.001**
Female	18	10 505	10 523	
Unknown	1	0	0	
Age, years				
< 72	21	9419	9440	**<0.001**
72 or Greater	24	8764	8788	
Unknown	1	0	0	
Race				
White	14	15 925	15 939	**<0.001**
Black	1	833	834	
Asian	21	1377	1398	
Unknown	10	48	58	
Tumor location				
Renal pelvis	26	11 978	12 004	0.182
Ureter	20	6205	6225	
Tumor focality				
Unifocal	43	13 374	13 417	**0.002**
Multifocal	3	4809	4812	
Tumor side				
Left	17	8986	9003	**<0.001**
Right	15	9044	9059	
Both	0	20	20	
Unknown	14	133	147	
pT stage				
T_is_–T_1_	3	1444	1447	**<0.001**
T_2_–T_4_	36	2716	2752	
Unknown	7	14 023	14 030	
Lymph node status				
Negative	22	3486	3508	**<0.001**
Positive	12	845	857	
Unknown	12	13 852	13 864	
Distant metastasis				
M_0_	21	3914	3935	**<0.001**
M_1_	0	659	659	
Unknown	25	13 610	13 635	
Type of surgery				
RNU	21	5477	5498	**<0.001**
RN	13	2503	2516	
Other types	7	2307	2314	
Non‐surgery	0	2267	2267	
Unknown	5	5629	5634	
Chemotherapy				
Yes	11	3206	3217	0.264
No/unknown	35	14 977	15 012	
Radiation				
Yes	4	1295	1299	0.679
No/unknown	42	16 888	16 930	

**Table 3:** Baseline characteristics of patients with lymphoepithelioma‐like carcinoma of the upper urinary tract and upper urinary tract urothelial carcinoma in 1 : 5 matched group. The bold values were applied to highlight *P*‐values, which had statistically significance (i.e., *P* < 0.05).

Characteristics	LELC group (*n* = 34)	UUT‐UC group (*n* = 166)	Total	*P*‐value
Gender				
Male	24	109	133	0.063
Female	9	57	66	
Unknown	1	0	1	
Age, years				
< 72	16	86	102	0.081
72 or Greater	17	80	97	
Unknown	1	0	0	
Race				
White	14	50	65	0.195
Black	1	25	26	
Asian	19	89	108	
Unknown	0	2	2	
Tumor location				
Renal pelvis	21	111	132	0.567
Ureter	13	55	68	
Tumor focality				
Unifocal	31	156	187	0.546
Multifocal	3	10	13	
Tumor side				
Left	17	83	100	**0.035**
Right	13	79	92	
Both	0	0	0	
Unknown	4	4	8	
pT stage				
T_is_‐T_1_	1	25	26	**0.016**
T_2_‐T_4_	26	84	110	
Unknown	7	57	64	
Lymph node status				
Negative	15	94	109	0.221
Positive	7	18	25	
Unknown	12	54	66	
Distant metastasis				
M_0_	19	94	113	0.091
M_1_	0	18	18	
Unknown	15	54	69	
Type of surgery				
RNU	18	82	98	0.180
RN	8	25	33	
Other types	5	24	29	
Non‐surgery	0	20	20	
Unknown	5	15	20	
Chemotherapy				
Yes	6	51	143	0.124
No/unknown	28	115	57	
Radiation				
Yes	4	20	24	0.963
No/unknown	30	146	176	

##### 3.4.1.2 Survival analyses

**Fig. 10:**
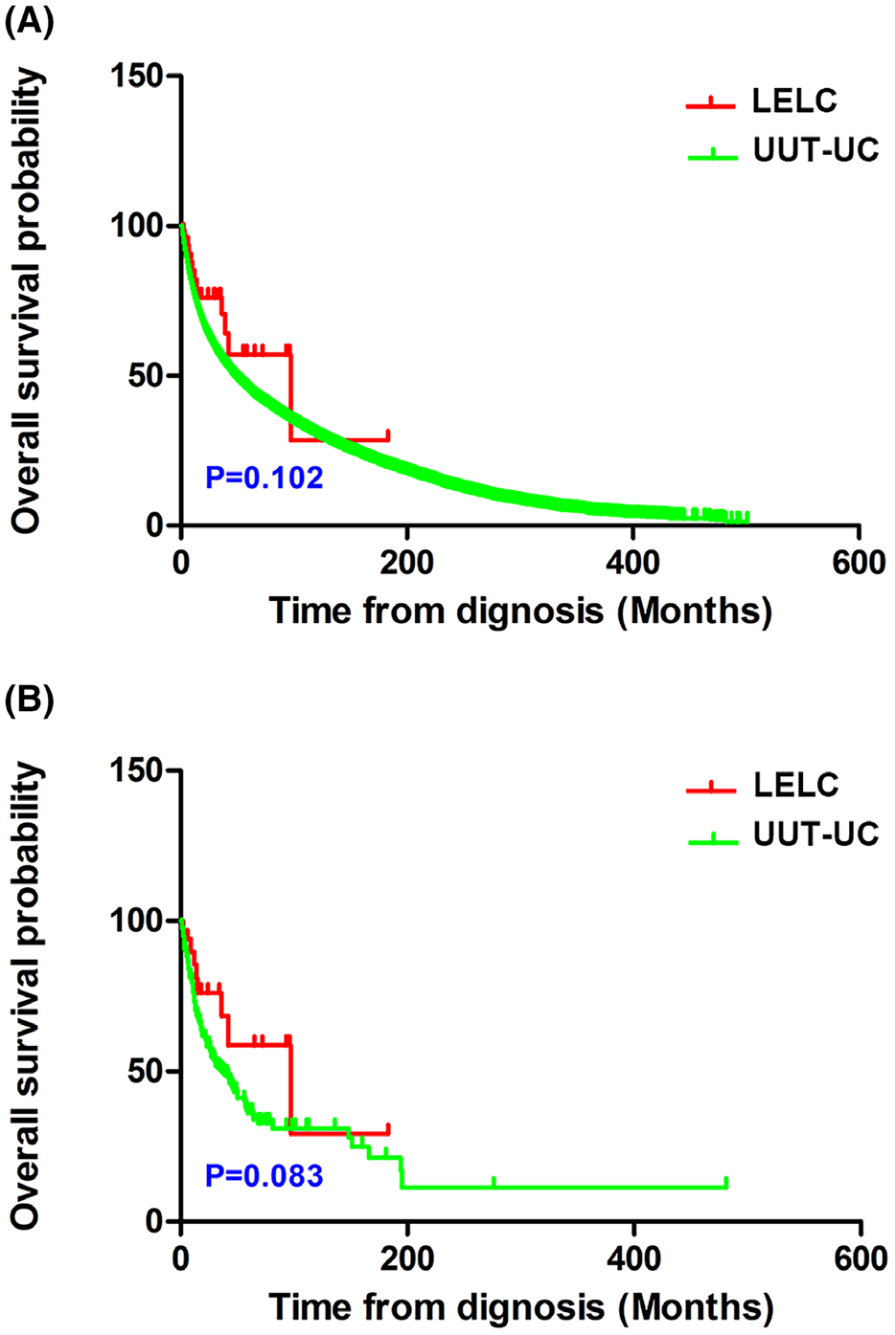
Overall survival curves between lymphoepithelioma‐like carcinoma (LELC) of the upper urinary tract and upper urinary tract urothelial carcinoma (UUT‐UC) group. (A) Before propensity score matching (LELC *n* = 46, UUT‐UC *n* = 18 183). (B) After propensity score matching (LELC *n* = 34, UUT‐UC *n* = 166). Data were analyzed by Kaplan–Meier method and were shown as mean ± SD.

The survival outcomes of patients with LELC and UUT‐UC are compared according to the Kaplan–Meier plots in Fig. 10. Overall, LELC of the upper urinary tract did not show significantly worse clinical outcomes than in UUT‐UC (Fig. 10A). Similarly, no significant difference in survival was observed between the two matching patient cohorts (Fig. 10B). The results indicate that patients with upper urinary tract urothelial carcinoma did not have significantly shortened survival compared with patients with lymphoepithelioma‐like carcinoma of the upper urinary tract.

#### 3.4.2 Identifying prognostic factors for LELC of the upper urinary tract

**Fig. 11:**
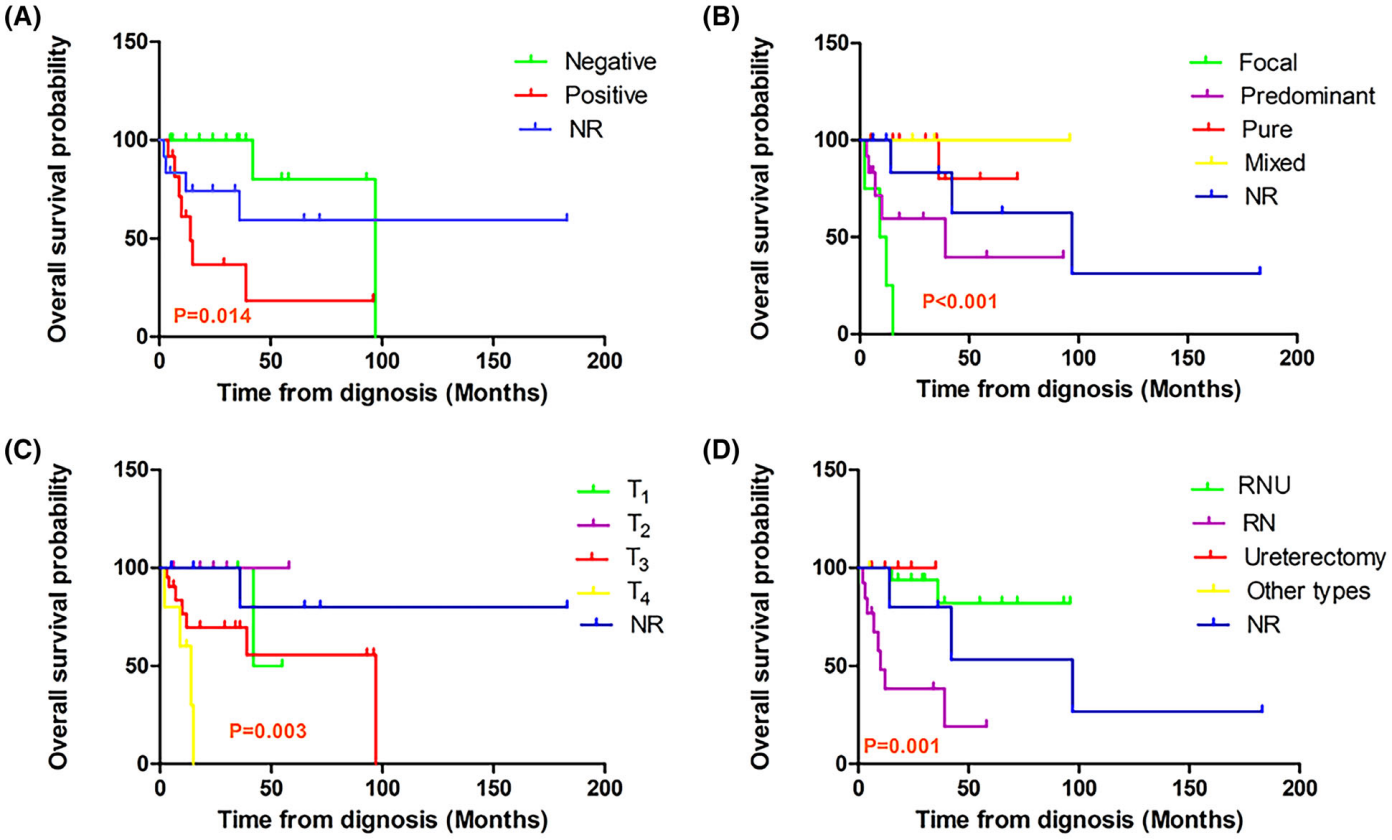
Overall survival of lymphoepithelioma‐like carcinoma (LELC) of the upper urinary tract patients. The images show the overall survival curves stratified by (A) lymph node status, (B) pathologic classification, (C) pathological stage, and (D) type of surgery in LELC patients (*n* = 46). NR, no reported; RN, radical nephrectomy; RNU, radical nephroureterectomy. Data were analyzed by Kaplan–Meier method and were shown as mean ± SD.

**Fig. 12:**
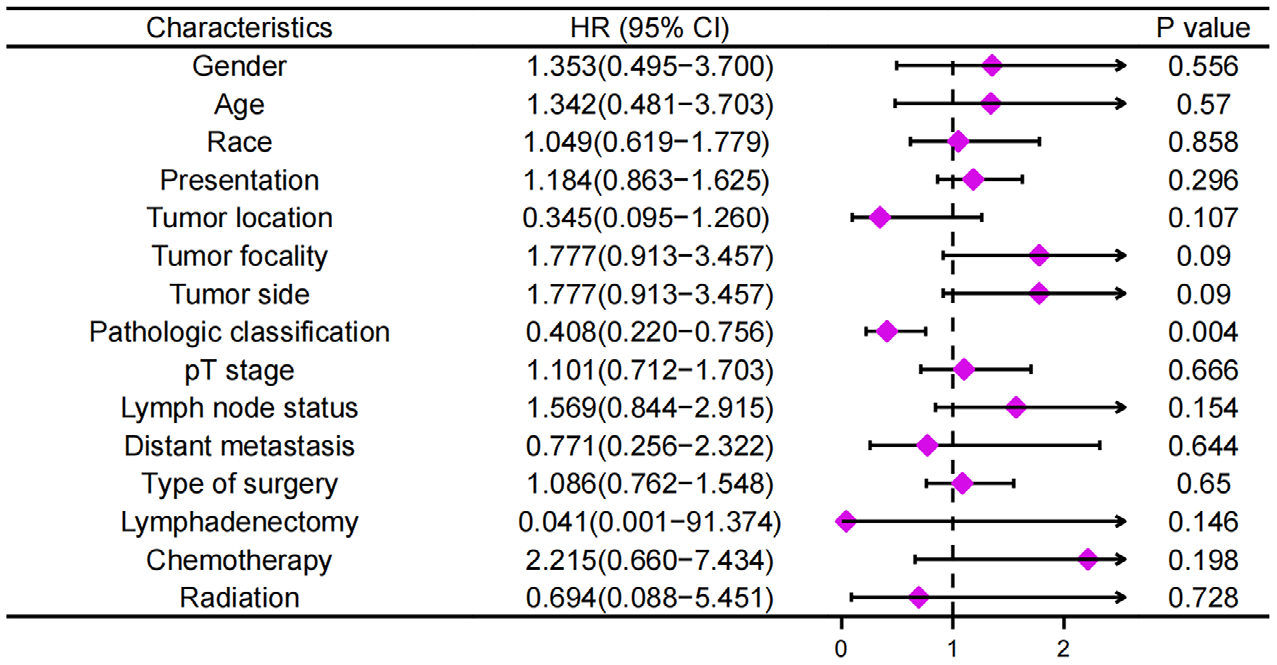
Forest plot of the univariate regression analysis revealing the relationship between different prognosis factors and overall survival in lymphoepithelioma‐like carcinoma (LELC) of the upper urinary tract patients (*n* = 46). The purple tetragonal diamonds represent the hazard ratios (HRs), and the horizontal line crossing the diamonds represents the 95% confidence intervals (95% CIs). Data were analyzed by Cox proportional hazards model method and were shown as HRs and 95% CI.

As shown in Table S12, the baseline characteristics of the samples are synthesized in numbers and percentages. Using Kaplan–Meier and univariate logistic regression analyses, potential prognostic factors have also been explored in patients with LELC of the upper urinary tract. In the Kaplan–Meier analysis, groups with negative lymph status (*P* = 0.014), pure pathological classification (*P* < 0.001), low pathologic stage (*P* = 0.003), and surgical treatment (*P* = 0.001) demonstrated higher overall cumulative survival rates (Fig. 11A–D). Furthermore, Fig. 12 presents the forest plots generated for the univariate analysis. After a univariate Cox regression analysis of initial factors associated with LELC prognosis, focal subtype was determined to have the potential to serve as a prognostic factor for overall survival in patients with LELC of the upper urinary tract (HR = 34.638, 95% CI = 3.708–323.562; *P* = 0.002; Table S13).

## 4 Discussion

The identification of susceptibility and driver genes through mutation analysis plays an integral role in the identification of clinically relevant genetic variations in patients with cancer. In the present study, one susceptibility gene (*KDM6A*) and one driver gene (*LEPR*) were validated in LELC using Sanger sequencing.

As a susceptibility gene verified by Sanger sequencing, *KDM6A* is a specific demethylase [60] that plays vital roles in early embryonic, cardiac, mammary, and immune tissue development [61]. Pernicious mutations in *KDM6A* are present in many cancer types, including urothelial carcinoma, bladder cancer, renal papillary cell carcinoma, some B/T‐cell lymphomas, and squamous cell carcinomas in the lung, head, and neck [20, 62, 63, 64, 65]. Kobatake et al. found that downregulated *KDM6A* expression could promote the polarization of M_2_ macrophages, increase tumor stem cells, and synergize with *p53* haploidy to lead to urothelial carcinoma. Low expression of *KDM6A* could reactively upregulate proinflammatory cytokines, including *CXCL1*, *CCL2*, and *IL6*, and then suppress urothelial cell growth [66]. Additionally, Kaneko *et al*. demonstrated that urothelium‐specific *KDM6A* downregulation increases the risk of bladder cancer in women. The loss of *KDM6A* can reduce the expression of certain cancer suppressor genes, such as *CDKN1A* and *PERP* [67]. Regarding clinical actionability, in multiple myeloma, *KDM6A* mutations accounted for 10% of 58 patients [68]. Another study showed that *KDM6A* was highly mutated in multiple cancer types, particularly bladder cancer, by sequencing genes from 4742 tumor samples from 21 cancer types [69]. Urothelial bladder carcinoma (UC) is the most common type of bladder cancer. Bladder cancer is one of the most common cancers in men in developed countries. Ler et al. [70] analyzed 176 urothelial bladder carcinoma samples by Sanger sequencing. They reported that the proportion of *KDM6A* mutations in non‐muscle‐invasive urothelial bladder carcinoma was 45%, muscle‐invasive tumors was 28%, and in tumors of unknown stages was 28%. Additionally, by searching and integrating other published data, they found that *KDM6A* mutations appeared in 29% of the urothelial bladder carcinoma samples.

Moreover, the encoding product of *LEPR* named the leptin receptor together with leptin maintains energy homeostasis and neuroendocrine function [71] and has been correlated with the occurrence and development of gastric, colorectal, and breast cancer [72, 73, 74]. Mutations in *LEPR* can result in obesity with additional features, such as severe obesity, alteration in immune function, hypogonadism, and hypothyroidism [71, 75, 76]. Furthermore, many SNPs in *LEPR* have been previously reported [77, 78]. Regarding clinical actionability, according to whole‐exome sequencing of hepatitis C virus (HCV)‐infected cirrhotic tissues, *LEPR* is one of the most common mutations in cirrhotic tissues, including tumor and nontumor tissues. Approximately, 57.1% *LEPR* mutations discovered in cirrhotic livers reduce *STAT3* phosphorylation, which can inactivate *LEPR*‐mediated signaling. Based on the analysis of liver tissue samples from patients with chronic HCV infection, *LEPR*‐induced somatic mutations accumulated in cirrhotic livers with chronic HCV infection. These mutations can cause *LEPR* signaling to break and increase susceptibility to hepatocarcinogenesis [79].

For clinically actionable, we had checked targeted mutations by reviewing the OncoKB website (https://www.oncokb.org/). We found that *KDM6A*, an X chromosome‐linked histone lysine demethylase, was frequently mutated in bladder cancer not only in European and American populations [80, 81], but also in Asian patients [82, 83]. Genetic alterations of *KDM6A* may be clinically actionable and related to the malignant progression of bladder cancer. Compelling biological evidence supports that tazemetostat may be effective in bladder cancer patients with *KDM6A* mutation. These evidences suggest that abnormalities and mutations in the susceptibility gene *KDM6A* and/or driver gene *LEPR* may be associated with case 2 of LELC of the renal pelvis. Our findings need to be validated by molecular biology and genetic studies based on primary cell culture in future. Meanwhile, the heterogeneity between different cases of LELC needs to be taken into account when applying the conclusions of hypothesis.

After analyzing the genetic characteristics of primary LELC of the upper urinary tract, 44 reported cases were collected of LELC of the upper urinary tract based on a comprehensive search of the SEER, PubMed, Medline, Cochrane, Web of Science, Embase, and Scopus databases, as well as combined with our two cases to comprise the LELC group. Meanwhile, 18 183 UUT‐UC data entries from the SEER database were used to compare patients with LELC in terms of prognostic risk factors and survival outcomes. The results demonstrated significant differences between the LELC and UUT‐UC groups in terms of baseline characteristics, including age (*P* < 0.001), sex (*P* < 0.001), and race (*P* < 0.001). In terms of clinical and pathological features, statistical differences were also detected in tumor focality (*P* = 0.002), tumor side (*P* < 0.001), pathological stage (*P* < 0.001), lymph node status (*P* < 0.001), distant metastasis (*P* < 0.001), and type of surgery (*P* < 0.001). According to the Kaplan–Meier survival curves, LELC did not indicate poorer overall survival than that for UUT‐UC. Nevertheless, we supposed that the differences mentioned above were not reliable owing to the marked impact of uneven baseline characteristics. Therefore, we performed propensity score matching in a proportion of 1 : 5, and 34 LELC cases were successfully matched with 166 UUT‐UC patients. After matching, the remaining significant differences included tumor side (*P* = 0.035) and pathological stage (*P* = 0.016). In addition, the overall survival was not significantly different between patients with LELC and UUT‐UC. The results indicate that patients with upper urinary tract urothelial carcinoma did not have a significantly shortened survival compared with patients with lymphoepithelioma‐like carcinoma of the upper urinary tract.

Lymphoepithelioma‐like carcinoma morphologically appears as nests, flakes, and strips with undifferentiated cell morphology, such as a large polymorphic nucleus, significant nucleolus, and ill‐defined intracellular boundary. As LELC of the upper urinary tract is rarely seen in clinical practice, it should be differentiated from tumor invasion into the upper urinary tract and from chronic inflammation of the upper urinary tract. Although invasive tumors contain neoplastic lymphocytes, the nuclei and cytoplasm of the epithelial cells are clearly defined. In contrast, in chronic inflammation, infiltrating cells in the mucosal layer and lamina propria are considered inflammatory cells [84, 85, 86]. Immunohistochemical staining showed tumor cells that were positive for CK 7, CK 8, CK 15, CK 19, CK AE1/AE3, and epithelial membrane antigen. Immunohistochemical analysis of lymphocytes in the tumor stroma typically detects positive staining for CD45, CD3, CD20, and CD138. Urothelial LELC is classified into three subtypes based on the percentage of lymphoepithelioma components [87]: (a) pure LELC refers to a single histologic subtype; (b) predominant LELC refers to a lymphoepithelial component greater than 50%, which is a dominant presence of LELC that is accompanied by typical urothelial carcinoma, squamous cell carcinoma, and/or adenocarcinoma; and (c) focal LELC refers to a lymphoepithelial component < 50%. Among the pathological classification evaluation of 46 reported cases of LELC of the upper urinary tract, including our two cases, 18 (31.5%) were pure LELCs, four (8.7%) were focal LELCs, and 12 (26.1%) were predominant LELCs. According to our Kaplan–Meier and Cox regression analyses, the focal subtype was determined to have the potential to serve as a prognostic factor for overall survival in patients with LELC of the upper urinary tract (HR = 34.638; *P* = 0.002).

Our study also has limitations. As LELC of the renal pelvis is very rare, the number of cases to date is insufficient to provide a reliable evidence and statistical conclusion. Furthermore, the limited samples for WGS made it difficult to completely avoid the false discovery rate associated with multiple testing effects, indicating that the proposed hypothesis in our study required further exploration of molecular mechanisms by biological or genetic validation. However, the isolation of primary cells from fresh tissues may be limited by the rarity or low incidence of LELC. In addition, immortalized human urothelial cells T24, 5637 or RT4 may not simulate the real malignant biological behavior of primary LELC cell. Meanwhile, single‐component cell line could not construct a real‐world tumor microenvironment. In the long run, the development of organ‐on‐a‐chip by microfluidic device or organoid technology may bring certain possibilities to solve the dilemma.

## 5 Conclusions

Lymphoepithelioma‐like carcinoma of the renal pelvis is a rare subtype of upper urinary tract carcinomas. This study presented patient‐specific characteristics, tumor‐specific features, potential mechanisms of pathogenesis, classification of LELC subtypes, possible prognoses, and therapeutic strategies. Awareness of this disease can help promote its early detection and diagnosis, prompt and effective treatment, and improve disease outcomes. Therefore, in cases of LELC of the renal pelvis, clinicians should ideally ascertain the biological behavior of the disease and arrive at a consensus on the best treatment options that would improve prognosis. To our knowledge, this is the first report to identify genetic information for LELC of the renal pelvis using WGS. Finally, it was found that mutations in the driver gene *LEPR* and susceptibility gene *KDM6A* may be associated with case 2 of LELC of the renal pelvis. These two genes may be involved in the metastasis and recurrence of tumors and provide a basis for clinical diagnosis and treatment. Our findings need to be validated by molecular biology and genetic studies based on primary cell culture in future. Meanwhile, the heterogeneity between different cases of LELC needs to be taken into account when applying the conclusions of hypothesis. Additionally, the prognosis of LELC of the upper urinary tract is similar to that of UUT‐UC. We suggest that the focal subtype can serve as a prognostic factor for LELC of the upper urinary tract, which warrants further studies.

## Conflict of interest

The authors declare no conflict of interest.

## Author contributions

BF, YH, HSZ, and XL conceived and designed this study. BF and XL undertook project leadership and guaranteed this work. ZL reviewed and guaranteed the ethical accuracy and feasibility of our study. BF, YH, HSZ, and TC performed the sequencing experiments. SW, HW, and TL carried out the immunohistochemical staining. BF, YH, HSZ, ST, XW, HXZ, and TH searched and collected the data from public databases. BF, YH, and TC analyzed and interpreted the data. BF, YH, HSZ, TC, and ZL wrote, reviewed, and revised the manuscript. All authors have read and approved the final version of this manuscript for publication.

## Supporting information


**Fig. S1.** Workflow diagram of the selection process for patients with lymphoepithelioma‐like carcinoma of the upper urinary tract.
**Fig. S2.** Somatic mutation heatmap of case 1 and 2.
**Fig. S3.** Frequency of mutated cancer cells.
**Table S1.** Antibodies used for immunohistochemistry.
**Table S2.** Number of SNPs in different regions of the genome and in coding regions.
**Table S3.** Number of SNP in different regions of the genome.
**Table S4.** Number of INDELs in different regions of the genome and in coding regions.
**Table S5.** Number of INDELs in different regions of the genome.
**Table S6.** Number of Somatic SNVs in different regions of the genome.
**Table S7.** Number of Somatic INDELs in different regions of the genome.
**Table S8.** Gene‐related structure variations discovered by whole‐genome sequencing in two cases.
**Table S9.** Detailed information regarding the tandem repeat regions identified in the primary lymphoepithelioma‐like carcinoma of the renal pelvis tissue in two cases.
**Table S10.** Analysis results of susceptibility genes in two cases.
**Table S11.** Analysis results of driving genes in two cases.
**Table S12.** Baseline demographic and clinicopathological characteristics of 46 patients with lymphoepithelioma‐like carcinoma of the upper urinary tract.
**Table S13.** Univariate regression analysis of pathologic classification associated with overall survival of patients with lymphoepithelioma‐like carcinoma of the upper urinary tract.

## Data Availability

Relevant research data have been presented in the text. All data will be provided upon request if necessary.
