# Supplementary material for: Analysis of genetic profiling, pathomics signature, and prognostic features of primary lymphoepithelioma‐like carcinoma of the renal pelvis
Source: Mol Oncol. 2022 Sep 2;16(20):3666–88. doi: 10.1002/1878-0261.13307 (PMC9580896; doi:10.1002/1878-0261.13307)
Supplement: Supplementary file 1 — Fig. S1. Workflow diagram of the selection process for patients with lymphoepithelioma‐like carcinoma of the upper urinary tract. Fig. S2. Somatic mutation heatmap of case 1 and 2. Fig. S3. Frequency of mutated cancer cells. Table S1. Antibodies used for immunohistochemistry. Table S2. Number of SNPs in different regions of the genome and in coding regions. Table S3. Number of SNP in different regions of the genome. Table S4. Number of INDELs in different regions of the genome and in coding regions. Table S5. Number of INDELs in different regions of the genome. Table S6. Number of Somatic SNVs in different regions of the genome. Table S7. Number of Somatic INDELs in different regions of the genome. Table S8. Gene‐related structure variations discovered by whole‐genome sequencing in two cases. Table S9. Detailed information regarding the tandem repeat regions identified in the primary lymphoepithelioma‐like carcinoma of the renal pelvis tissue in two cases. Table S10. Analysis results of susceptibility genes in two cases. Table S11. Analysis results of driving genes in two cases. Table S12. Baseline demographic and clinicopathological characteristics of 46 patients with lymphoepithelioma‐like carcinoma of the upper urinary tract. Table S13. Univariate regression analysis of pathologic classification associated with overall survival of patients with lymphoepithelioma‐like carcinoma of the upper urinary tract. [file MOL2-16-3666-s001.docx]

**Supplementary materials**

**Supplementary tables**

**Table S1.** Antibodies used in the immunohistochemistry. GATA3, GATA binding protein 3; p63, protein-63; CD, cluster of differentiation.

| **Antibody** | **Species** | **Antibody number** | **Source** | **Dilution** |
| --- | --- | --- | --- | --- |
| Ki67 | Rabbit | 27309-1-AP | Protein Tech Group, Chicago, USA | 1:6000 |
| GATA3 | Rabbit | ab199428 | Abcam, Cambridge, UK | 1:500 |
| p63 | Mouse | ab735 | Abcam, Cambridge, UK | 1:100 |
| CD3 | Rabbit | ab16669 | Abcam, Cambridge, UK | 1:100 |
| CD10 | Rabbit | ab256494 | Abcam, Cambridge, UK | 1:500 |
| CD20 | Rabbit | ab78237 | Abcam, Cambridge, UK | 1:100 |
| CD45 | Rabbit | ab40763 | Abcam, Cambridge, UK | 1:400 |
| CD68 | Mouse | ab955 | Abcam, Cambridge, UK | 1:200 |
| CD163 | Rabbit | ab182422 | Abcam, Cambridge, UK | 1:400 |
| Cytokeratin AE1/AE3 | Mouse | ab27988 | Abcam, Cambridge, UK | 1:20 |
| Cytokeratin  34βE12 | Mouse | ab776 | Abcam, Cambridge, UK | 1:50 |
| Cytokeratin 7 | Rabbit | ab68459 | Abcam, Cambridge, UK | 1:1000 |
| Cytokeratin 20 | Rabbit | ab76126 | Abcam, Cambridge, UK | 1:200 |

**Table S2.** The number of SNPs in different regions of the genome and in coding regions. SNPs, single nucleotide polymorphisms; LELC, lymphoepithelioma-like carcinoma; UTR, untranslated region; ncRNA, noncoding ribonucleic acid.

| **Sample** | **Case 1** | |  | **Case 2** | |
| --- | --- | --- | --- | --- | --- |
|  | **LELC specimen** | **Adjacent specimen** |  | **LELC specimen** | **Adjacent specimen** |
| CDS | 19,233 | 16,936 |  | 5,752 | 10,162 |
| Synonymous SNP | 9,923 | 8,796 |  | 2,635 | 5,083 |
| Missense SNP | 8,887 | 7,788 |  | 2,938 | 4,825 |
| Stopgain | 62 | 55 |  | 76 | 60 |
| Stoploss | 5 | 5 |  | 3 | 8 |
| Unknown | 356 | 292 |  | 100 | 186 |
| Intronic | 1,125,097 | 991,426 |  | 615,358 | 924,340 |
| UTR3 | 22,039 | 18,302 |  | 7,988 | 14,954 |
| UTR5 | 4,631 | 4,326 |  | 1,109 | 1,668 |
| Splicing | 499 | 447 |  | 170 | 280 |
| ncRNA exonic | 8,398 | 7,006 |  | 3,040 | 5,288 |
| ncRNA intronic | 132,066 | 112,722 |  | 68,626 | 108,578 |
| ncRNA splicing | 102 | 77 |  | 36 | 74 |
| Upstream | 18,356 | 16,224 |  | 7,365 | 10,887 |
| Downstream | 19,715 | 17,111 |  | 9,865 | 14,617 |
| Intergenic | 1,917,871 | 1,636,540 |  | 1,034,848 | 1,616,845 |
| Others | 631 | 605 |  | 267 | 335 |
| Total | 3,268,638 | 2,821,722 |  | 1,754,424 | 2,708,028 |

**Table S3.** The number of SNP in different regions of the genome. SNP, single nucleotide polymorphism; LELC, lymphoepithelioma-like carcinoma; TS, transformation; TV, transmutation; dbSNP, the single nucleotide polymorphism.

| **Sample** | **Case 1** | |  | **Case 2** | |
| --- | --- | --- | --- | --- | --- |
|  | **LELC specimen** | **Adjacent specimen** |  | **LELC specimen** | **Adjacent specimen** |
| Total | 3,268,638 | 2,821,722 |  | 1,754,424 | 2,708,028 |
| Het | 1,692,935 | 1,565,626 |  | 1,151,719 | 1,529,120 |
| Hom | 1,575,703 | 1,256,096 |  | 602,705 | 1,178,908 |
| Transition | 2,209,476 | 1,910,767 |  | 1,223,653 | 1,836,824 |
| Transvertion | 1,059,162 | 910,955 |  | 530,771 | 871,204 |
| TS/TV | 2.09 | 2.10 |  | 2.31 | 2.11 |
| dbSNP percentage | 3,243,976 (99.25%) | 2,764,769 (97.98%) |  | 1,332,256 (75.94%) | 2,590,173 (95.65%) |
| Novel | 24,662 | 56,953 |  | 422,168 | 117,855 |
| Novel TS | 15,116 | 37,800 |  | 304,424 | 84,248 |
| Novel TV | 9,546 | 19,153 |  | 117,744 | 33,607 |
| Novel TS/TV | 1.58 | 1.97 |  | 2.59 | 2.51 |

**Table S4.** The number of INDELs in different regions of the genome and in coding regions. INDELs, insertions and deletions; LELC, lymphoepithelioma-like carcinoma; CDS, coding sequence; UTR, untranslated region; ncRNA, noncoding ribonucleic acid.

| **Sample** | **Case 1** | |  | **Case 2** | |
| --- | --- | --- | --- | --- | --- |
|  | **LELC specimen** | **Adjacent specimen** |  | **LELC specimen** | **Adjacent specimen** |
| CDS | 503 | 525 |  | 415 | 324 |
| Frameshift deletion | 68 | 94 |  | 150 | 78 |
| Frameshift insertion | 44 | 35 |  | 46 | 27 |
| Nonframeshift deletion | 182 | 200 |  | 145 | 114 |
| Nonframeshift insertion | 133 | 123 |  | 43 | 75 |
| Stopgain | 2 | 4 |  | 6 | 1 |
| Unknown | 74 | 69 |  | 25 | 29 |
| Intronic | 187,177 | 152,226 |  | 97,125 | 148,058 |
| UTR3 | 4,326 | 3,173 |  | 1,540 | 2,889 |
| UTR5 | 606 | 582 |  | 157 | 231 |
| Splicing | 124 | 99 |  | 51 | 73 |
| ncRNA exonic | 961 | 789 |  | 396 | 617 |
| ncRNA intronic | 21,835 | 16,814 |  | 10,719 | 16,962 |
| ncRNA UTR5 | 2 | 1 |  | 1 | 1 |
| ncRNA splicing | 15 | 7 |  | 5 | 8 |
| Upstream | 3,462 | 2,897 |  | 1,522 | 2,078 |
| Downstream | 3,720 | 3,066 |  | 1,787 | 2,734 |
| Intergenic  Others | 300,582  104 | 233,921  98 |  | 153,255  32 | 238,857  60 |
| Total | 523,417 | 414,198 |  | 267,005 | 412,892 |

**Table S5.** The number of INDELs in different regions of the genome. INDELs, insertions and deletions; LELC, lymphoepithelioma-like carcinoma; dbSNP, the single nucleotide polymorphism.

| **Sample** | **Case 1** | |  | **Case 2** | |
| --- | --- | --- | --- | --- | --- |
|  | **LELC specimen** | **Adjacent specimen** |  | **LELC specimen** | **Adjacent specimen** |
| Total  Het  Hom  dbSNP percentage  Novel | 523,417  234,904  288,513  469,351 (89.67%)  54,066 | 414,198  201,520  212.678  363,573 (87.78%）  50,625 |  | 267,005  143,782  123,223  185,716 (69.56%)  81,289 | 412,892  196,903  215,989  349,300 (84.60%)  63,592 |

**Table S6.** The number of Somatic SNVs in different regions of the genome. SNVs, single nucleotide variants; CDS, coding sequence; SNP, single nucleotide polymorphism; UTR, untranslated region; ncRNA, noncoding ribonucleic acid.

| **Sample** | **Case 1** |  | **Case 2** |
| --- | --- | --- | --- |
|  | **LELC specimen** |  | **LELC specimen** |
| CDS | 102 |  | 1 |
| Synonymous SNP | 31 |  | 1 |
| Missense SNP | 60 |  | 0 |
| Stopgain | 7 |  | 0 |
| Unknown | 4 |  | 0 |
| Intronic | 3314 |  | 979 |
| UTR3 | 63 |  | 7 |
| UTR5 | 35 |  | 2 |
| Splicing | 2 |  | 1 |
| ncRNA exonic | 41 |  | 2 |
| ncRNA intronic | 528 |  | 102 |
| ncRNA splicing | 1 |  | 0 |
| Upstream | 60 |  | 10 |
| Downstream | 62 |  | 18 |
| Intergenic | 5901 |  | 1559 |
| Others | 1 |  | 1 |
| Total | 10110 |  | 2682 |

**Table S7.** The number of Somatic INDELs in different regions of the genome. INDELs, insertions and deletions; LELC, lymphoepithelioma-like carcinoma; ncRNA, noncoding ribonucleic acid.

| **Sample** | **Case 1** |  | **Case 2** |
| --- | --- | --- | --- |
|  | **LELC specimen** |  | **LELC specimen** |
| Intronic | 25 |  | 435 |
| UTR3 | 1 |  | 3 |
| ncRNA intronic | 3 |  | 46 |
| Upstream | 0 |  | 3 |
| Downstream | 1 |  | 8 |
| Intergenic | 33 |  | 711 |
| Total | 63 |  | 1206 |

**Table S8.** Gene-related structure variations discovered by whole-genome sequencing in two cases. FPG, fusion partner gene; CDS, coding sequence.

| **Type** | **Breakpoint A** | **Breakpoint B** | **5_FPG_GENE_NAME** | **5_IN_CDS?** | **5_SEGMENT_TYPE** | **3_FPG_GENE_NAME** | **3_IN_CDS?** | **3_SEGMENT_TYPE** |
| --- | --- | --- | --- | --- | --- | --- | --- | --- |
| Translocation | chr12:56596312 | chr15:39702424 | *BAZ2A* | No | Intron | *FSIP1* | Yes | Intron |

**Table S9.** Detailed information regarding the tandem repeat regions identified in the primary LELC of the renal pelvis tissue in two cases. Trf, transformation; INDEL, insertions and deletion; Chr, chromosome; ncRNA, noncoding ribonucleic acid.

| **Chrom** | **Trf_**  **Start** | **Trf_**  **End** | **Trf_**  **Score** | **Trf_**  **repeat_period** | **Trf_**  **repeat_times** | **Trf_repeat_**  **\consensus** | **INDEL_region** | **Gene** |
| --- | --- | --- | --- | --- | --- | --- | --- | --- |
| Chr16 | 64629641 | 64629684 | 52 | 15 | 3 | AAACCAGCTAAACC | Intergenic | *NONE*;*CDH11* |
| Chr 8 | 67530614 | 67530681 | 66 | 5 | 13.6 | ATGGA | Intronic | *CPA6* |
| Chr10 | 49785266 | 49785301 | 54 | 15 | 2.4 | ACCACCACCACCATC | Intergenic | *OGDHL*;*PARG* |
| Chr14 | 68070250 | 68070279 | 60 | 4 | 7.5 | TATC | Intronic | *RAD51B* |
| Chr Y | 22359895 | 22359943 | 98 | 4 | 12.2 | CTTT | Intronic | *RBMY1F* |
| Chr 4 | 183770448 | 183770503 | 76 | 12 | 4.5 | ATATGGATACAT | Intergenic | *TRAPPC11*;*NONE* |
| Chr 1 | 166848565 | 166848595 | 62 | 2 | 15.5 | CA | Intronic | *POGK* |
| Chr 4 | 8541638 | 8541668 | 62 | 2 | 15.5 | GT | Intergenic | *TRMT44*;*GPR78* |
| Chr 1 | 108641545 | 108641572 | 56 | 2 | 14 | GT | Intergenic | *FAM102B*;*HENMT1* |
| Chr 2 | 182545086 | 182545131 | 74 | 5 | 9.2 | ATGGA | Intergenic | *PDE1A*;*DNAJC10* |
| Chr 8 | 101369843 | 101369873 | 62 | 4 | 7.8 | GCCT | Downstream | *NACAP1* |
| Chr19 | 2833464 | 2833497 | 50 | 4 | 8.2 | CTTT | Intronic | *ZNF554* |
| Chr 9 | 37915231 | 37915290 | 61 | 4 | 14.8 | CTTT | Downstream | *SHB* |
| Chr13 | 73215742 | 73215771 | 51 | 9 | 3.2 | TTTTTTCTT | Intergenic | *KLF5*;*LINC00392* |
| Chr 8 | 126284563 | 126284596 | 50 | 16 | 2.1 | CTGTCTGTCTCTCTCT | Intergenic | *LINC00861*;*LOC101927657* |
| Chr 9 | 38308323 | 38308356 | 52 | 4 | 8.8 | GATA | Intergenic | *SHB*;*ALDH1B1* |
| Chr 5 | 154986470 | 154986499 | 51 | 15 | 2.1 | TGTGTTGTGTTGTA | Intergenic | *MRPL22*;*KIF4B* |
| Chr 1 | 64229167 | 64229192 | 52 | 3 | 8.7 | AAC | Intronic | *UBE2U* |
| Chr16 | 87033727 | 87033766 | 53 | 5 | 8 | TCCAT | Intergenic | *LINC02188*;*LINC02181* |
| Chr11 | 9169500 | 9169529 | 60 | 2 | 15 | CA | Intronic | *DENND5A* |
| Chr 9 | 30719810 | 30719859 | 55 | 20 | 2.5 | ATTTCATTCCATACCATTTG | Intergenic | *LINC01242*;*LINC01243* |
| Chr 4 | 104848580 | 104848624 | 63 | 19 | 2.5 | TAAAGTTTTTTTGTAAA | Intergenic | *CXXC4-AS1*;*TET2* |
| Chr 8 | 145011655 | 145011700 | 85 | 4 | 11.8 | TTTC | Intergenic | *ZNF252P-AS1*;*C8orf33* |
| Chr13 | 28914308 | 28914350 | 68 | 22 | 2 | TCTCTCTCCCTCCCTTTCTGTT | Intergenic | *SLC46A3*;*MTUS2* |
| Chr 4 | 95309279 | 95309329 | 93 | 18 | 2.8 | GACCCTAACCACCACCAT | Intronic | *UNC5C* |
| Chr16 | 80303593 | 80303626 | 52 | 15 | 2.2 | TCTTTCTTTCTTCCTT | ncRNA_intronic | *LOC102724084* |
| Chr 6 | 2852895 | 2852969 | 141 | 38 | 2 | TGTTCACACAGTGCGGTGCTGACTCTCTGTATGAAGCG | Intergenic | *SERPINB1*;*MIR4645* |
| Chr13 | 40212380 | 40212412 | 52 | 18 | 1.9 | CTTTTTTCTTTTCTTTTC | ncRNA_intronic | *LINC00548* |
| Chr 8 | 18867607 | 18867670 | 128 | 31 | 2.1 | TCATCTCTCCCTCTCCCCAGAGACACCCACA | Intronic | *PSD3* |
| Chr X | 118079653 | 118079699 | 58 | 13 | 3.4 | CACACACATACAC | Intronic | *KLHL13* |
| Chr 6 | 118944257 | 118944301 | 90 | 15 | 3 | ACCCTCTCCCCACAC | Intergenic | *MCM9*;*FAM184A* |
| Chr11 | 68336787 | 68336816 | 51 | 16 | 1.9 | TTTATAGTTTTTTTT | Intronic | *LRP5* |
| Chr 6 | 7871260 | 7871318 | 54 | 2 | 31.5 | TG | Intronic | *BMP6* |
| Chr10 | 10144336 | 10144376 | 68 | 16 | 2.7 | TACATACACACACACC | Intergenic | *LOC101928298*;*LOC101928322* |
| Chr13 | 61606921 | 61606945 | 50 | 2 | 12.5 | AC | Intergenic | *LINC02339*;*LINC00358* |
| Chr 4 | 46273487 | 46273536 | 82 | 2 | 25 | AT | Intronic | *GABRA2* |
| Chr14 | 45124541 | 45124573 | 59 | 6 | 5.7 | AAAAGA | Intronic | *FKBP3* |
| Chr 5 | 99500599 | 99500651 | 56 | 5 | 10.8 | TTGTA | Intergenic | *LOC100289230*;*LINC02113* |
| Chr 6 | 4614205 | 4614265 | 65 | 4 | 15.5 | TCTT | Intergenic | *KU-MEL-3*;*CDYL* |
| Chr 4 | 60159384 | 60159420 | 74 | 16 | 2.3 | CCCTTTCCTACACCAC | Intergenic | *LINC02429*;*MIR548AG1* |
| Chr 2 | 76602026 | 76602069 | 54 | 5 | 8.8 | CTCCA | Intergenic | *GCFC2*;*LRRTM4* |
| Chr 2 | 219404409 | 219404447 | 55 | 5 | 8.2 | AAACA | Intergenic | *DNPEP*;*DES* |
| Chr 8 | 35179568 | 35179618 | 61 | 16 | 2.9 | TGTGTATGTGTATATATA | Intergenic | *LINC01288*;*UNC5D* |
| Chr 4 | 73076810 | 73076884 | 78 | 5 | 15 | TGGAA | Intronic | *ANKRD17* |
| Chr 9 | 117484049 | 117484108 | 66 | 12 | 4.8 | GTGTGTGGGCGT | Intergenic | *ASTN2*;*LOC101928797* |
| Chr17 | 10480411 | 10480441 | 53 | 12 | 2.6 | TGTGTGTATATA | ncRNA_intronic | *MYHAS* |
| Chr14 | 49719742 | 49719798 | 96 | 5 | 11.4 | ATTGT | Intronic | *KLHDC1* |
| Chr 3 | 197271233 | 197271262 | 60 | 2 | 15 | AC | Intronic | *DLG1* |
| Chr 3 | 17724792 | 17724826 | 70 | 2 | 17.5 | GT | Intronic | *TBC1D5* |
| Chr14 | 99385507 | 99385554 | 51 | 20 | 2.4 | CTGTCTGTCCCTCTCTCTCT | Intergenic | *BCL11B*;*SETD3* |
| Chr 7 | 14789945 | 14789978 | 50 | 16 | 2.1 | TGTCTCTCTCTCTCTC | Intronic | *DGKB* |
| Chr 6 | 35630560 | 35630584 | 50 | 12 | 2.1 | AAAGAAAAGAAA | Intronic | *FKBP5* |
| Chr 3 | 56995645 | 56995671 | 54 | 2 | 13.5 | GA | Intronic | *ARHGEF3* |
| Chr12 | 104811568 | 104811614 | 67 | 5 | 9.4 | ATGGA | Intronic | *SLC41A2* |
| Chr11 | 71393635 | 71393670 | 63 | 15 | 2.4 | CCATCATCACCATCA | Intergenic | *SHANK2*;*FLJ42102* |
| Chr21 | 45713026 | 45713062 | 58 | 14 | 2.6 | TTTATTTATTTTAT | Intronic | *PCBP3* |
| Chr14 | 45433617 | 45433671 | 85 | 18 | 3.1 | 3.1 | Intergenic | *MIS18BP1*;*LINC02303* |
| Chr19 | 42506069 | 42506111 | 59 | 15 | 2.9 | AATGGACACGAATGG | ncRNA_intronic | *LIPE-AS1* |
| Chr X | 54618427 | 54618474 | 51 | 3 | 15.3 | CTT | Intergenic | *GNL3L*;*ITIH6* |
| Chr X | 133667919 | 133667946 | 56 | 14 | 2 | TCTTTCTTTCTTTT | Intronic | *GPC3* |
| Chr 9 | 117179365 | 117179389 | 50 | 5 | 5 | TTTTG | Intronic | *ASTN2* |
| Chr13 | 108058583 | 108058618 | 54 | 17 | 2.1 | AGGAGAGAGAGAGAGAG | Intergenic | *FAM155A*;*LIG4* |
| Chr 1 | 68599594 | 68599624 | 53 | 16 | 1.9 | TTAATTTTACAAAATT | Intergenic | *DEPDC1-AS1*;*LINC01707* |
| Chr18 | 26954861 | 26954888 | 56 | 4 | 7 | TTTG | Intronic | *CHST9* |
| Chr 4 | 38245621 | 38245670 | 61 | 21 | 2.4 | CACCACCACTACCACCATCACT | Intergenic | *TBC1D1*;*LINC01258* |
| Chr 4 | 73134351 | 73134379 | 58 | 9 | 3.2 | CCACCATCA | Intronic | *ANKRD17* |
| Chr 4 | 64807158 | 64807189 | 55 | 15 | 2.1 | TCTCTTCTTCTCTCT | Intergenic | *TECRL*;*LINC02232* |
| Chr 2 | 2181202 | 2181237 | 54 | 12 | 3 | GTGTACCTGTGT | Intronic | *MYT1L* |
| Chr15 | 90259387 | 90259425 | 62 | 16 | 2.4 | AAGGAAGAAAGGAAAGG | Downstream | *TTLL13P* |
| Chr 8 | 30214396 | 30214434 | 53 | 12 | 3.5 | GTTTTTTGTTT | Intergenic | *DCTN6*;*RBPMS-AS1* |
| Chr16 | 132313 | 132339 | 54 | 6 | 4.5 | TTTATT | Intronic | *NPRL3* |
| Chr 7 | 130150019 | 130150056 | 58 | 18 | 2.2 | TCTGTGCCTATCTTCTG | Intergenic | *KLHDC10*;*TMEM209* |
| Chr14 | 82566338 | 82566368 | 55 | 8 | 4 | TGTTTTTT | Intergenic | *LINC02311*;*LINC02301* |
| Chr 2 | 34813860 | 34813907 | 96 | 2 | 24 | TA | Intergenic | *LINC01320*;*MIR548AD* |
| Chr16 | 79550053 | 79550086 | 50 | 10 | 3.4 | CTCTGTCTGT | Intergenic | *WWOX*;*MAF* |
| Chr 7 | 27622886 | 27622932 | 67 | 15 | 3.1 | AATGGAATGGAATCG | ncRNA_intronic | *TSL* |
| Chr 4 | 170622988 | 170623021 | 50 | 5 | 6.8 | TGGAG | Intergenic | *LINC01612*;*LINC02382* |
| Chr 5 | 109887787 | 109887816 | 51 | 15 | 2 | TTTTTGCATTCCATT | Intergenic | *LINC01848*;*TMEM232* |
| Chr 1 | 55928560 | 55928592 | 57 | 11 | 3 | TGTGTGTGATG | ncRNA_intronic | *LINC01753* |

**Table S10.** Analysis results of susceptibility genes in two cases. Ref, reference; Alt, alternative; AA, amino acid.

| **Hugo**  **Symbol** | **Chromosome** | **Position** | | **Ref**  **allele** | **Alt**  **allele** | **Variant Classification** | **AAChange** |
| --- | --- | --- | --- | --- | --- | --- | --- |
| *TP53BP1* | 15 | 43705415 | C | | A | Missense_Mutation | *TP53BP1*:NM_001141979:exon24:c.G5201T:p.G1734V\|*TP53BP1*:NM_001141980:exon24:c.G5207T:p.G1736V\|*TP53BP1*:NM_005657:exon24:c.G5192T:p.G1731V |
| *CLSPN* | 1 | 36219528 | G | | T | Missense_Mutation | *CLSPN*:NM_022111:exon9:c.C1589A:p.A530D |
| *HOXA11* | 7 | 27224528 | A | | G | Missense_Mutation | *HOXA11*:NM_005523:exon1:c.T236C:p.L79P |
| *KDM6A* | X | 44928980 | G | | A | Missense_Mutation | *KDM6A*:NM_021140:exon17:c.G2080A:p.A694T |
| *TP53BP1* | 15 | 43712554 | G | | A | Missense_Mutation | *TP53BP1*:NM_001141979:exon21:c.C4630T:p.P1544S\|*TP53BP1*:NM_001141980:exon21:c.C4630T:p.P1544S\|*TP53BP1*:NM_005657:exon21:c.C4615T:p.P1539S |
| *POLE* | 12 | 133249269 | C | | T | Missense_Mutation | *POLE*:NM_006231:exon15:c.G1630A:p.V544M |
| *PLXNA1* | 3 | 126707950 | G | | A | Missense_Mutation | *PLXNA1*:NM_032242:exon1:c.G514A:p.V172M |
| *MYO5A* | 15 | 52708421 | C | | T | Missense_Mutation | *MYO5A*:NM_000259:exon5:c.G533A:p.R178Q\|  *MYO5A*:NM_001142495:exon5:c.G533A:p.R178Q |
| *MDM2* | 12 | 69233525 | C | | A | Missense_Mutation | *MDM2*:NM_001145340:exon5:c.C784A:p.L262I\|  *MDM2*:NM_001278462:exon6:c.C862A:p.L288I\|  *MDM2*:NM_001145339:exon9:c.C1225A:p.L409I\|  *MDM2*:NM_001145337:exon11:c.C1231A:p.L411I\|  *MDM2*:NM_002392:exon11:c.C1390A:p.L464I |
| *MN1* | 22 | 28196290 | C | | A | Missense_Mutation | *MN1*:NM_002430:exon1:c.G242T:p.G81V |
| *HIP1* | 7 | 75174045 | A | | G | Missense_Mutation | *HIP1*:NM_001243198:exon25:c.T2561C:p.V854A\|  *HIP1*:NM_005338:exon27:c.T2714C:p.V905A |
| *KMT2C* | 7 | 151856028 | G | | A | Missense_Mutation | *KMT2C*:NM_170606:exon44:c.C11590T:p.R3864C |
| *IREB2* | 15 | 78758642 | G | | A | Missense_Mutation | *IREB2*:NM_004136:exon5:c.G440A:p.G147D |
| *MACF1* | 1 | 39888178 | A | | T | Missense_Mutation | *MACF1*:NM_012090:exon53:c.A9763T:p.N3255Y |
| *NUP98* | 11 | 3712598 | A | | G | Missense_Mutation | *NUP98*:NM_016320:exon28:c.T4433C:p.L1478P\|  *NUP98*:NM_139132:exon28:c.T4433C:p.L1478P |
| *PSIP1* | 9 | 15469314 | G | | T | Missense_Mutation | *PSIP1*:NM_001128217:exon12:c.C1054A:p.L352I\|  *PSIP1*:NM_033222:exon12:c.C1054A:p.L352I |
| *TRERF1* | 6 | 42214282 | C | | T | Missense_Mutation | *TRERF1*:NM_033502:exon14:c.G2657A:p.G886D |
| *BRWD1* | 21 | 40642232 | C | | T | Missense_Mutation | *BRWD1*:NM_018963:exon14:c.G1372A:p.G458R\|  *BRWD1*:NM_033656:exon14:c.G1372A:p.G458R |
| *SLC45A3* | 1 | 205633628 | T | | A | Missense_Mutation | *SLC45A3*:NM_033102:exon2:c.A157T:p.M53L |
| *APC* | 5 | 112174669 | C | | G | Missense_Mutation | *APC*:NM_001127511:exon14:c.C3324G:p.S1108R\|  *APC*:NM_000038:exon16:c.C3378G:p.S1126R\|  *APC*:NM_001127510:exon17:c.C3378G:p.S1126R |

**Table S11.** Analysis results of driving genes in two cases. Ref, reference; Alt, alternative; AA, amino acid.

| **Hugo**  **Symbol** | **Chromosome** | **Position** | **Ref**  **allele** | **Alt**  **allele** | **Variant Classification** | **AAChange** |
| --- | --- | --- | --- | --- | --- | --- |
| *LEPR* | 1 | 66096026 | C | T | Missense_Mutation | *LEPR*:NM_001198687:exon19:c.C2815T:p.L939F\|  *LEPR*:NM_001003680:exon20:c.C2815T:p.L939F |
| *HMGA1* | 6 | 34208631 | G | A | Missense_Mutation | *HMGA1*:NM_145901:exon2:c.G74A:p.G25D\|  *HMGA1*:NM_145902:exon2:c.G74A:p.G25D\|  *HMGA1*:NM_145903:exon2:c.G74A:p.G25D\|  *HMGA1*:NM_145905:exon2:c.G74A:p.G25D\|  *HMGA1*:NM_002131:exon3:c.G74A:p.G25D\|  *HMGA1*:NM_145899:exon3:c.G74A:p.G25D |
| *CNOT1* | 16 | 58633241 | T | C | Missense_Mutation | *CNOT1*:NM_001265612:exon2:c.A1G:p.M1V\|  *CNOT1*:NM_016284:exon2:c.A1G:p.M1V\|  *CNOT1*:NM_206999:exon2:c.A1G:p.M1V |
| *EPPK1* | 8 | 1.45E+08 | C | G | Missense_Mutation | *EPPK1*:NM_031308:exon2:c.G7132C:p.D2378H |

**Table S12.** Baseline demographic and clinicopathological characteristics of 46 patients with lymphoepithelioma-like carcinoma of the upper urinary tract. RNU, radical nephroureterectomy; RN, radical nephrectomy.

| **Characteristics** | **Numbers**  **(n)** | **Percentage**  **(%)** |
| --- | --- | --- |
| Gender |  |  |
| Male | 27 | 58.7 |
| Female | 18 | 391 |
| Unknown | 1 | 0.02 |
| Age, years |  |  |
| Less than 72 | 21 | 45.7 |
| 72 or Greater | 24 | 52.2 |
| Unknown | 1 | 0.02 |
| Race |  |  |
| White | 14 | 30.4 |
| Black | 1 | 0.02 |
| Asian | 21 | 45.7 |
| Unknown | 10 | 21.7 |
| Tumor location |  |  |
| Renal pelvis | 26 | 56.5 |
| Ureter | 20 | 43.5 |
| Tumor focality |  |  |
| Unifocal | 43 | 93.5 |
| Multifocal | 3 | 6.5 |
| Tumor side |  |  |
| Left | 17 | 37.0 |
| Right | 15 | 32.6 |
| Unknown | 14 | 30.4 |
| Pathologic classification |  |  |
| Pure subtype | 18 | 39.1 |
| Focal subtype | 4 | 8.7 |
| Predominant subtype | 12 | 26.1 |
| Mixed subtype | 3 | 6.5 |
| Unknown | 9 | 20.0 |
| pT stage |  |  |
| T_1_ | 3 | 6.5 |
| T_2_ | 10 | 21.7 |
| T_3_ | 21 | 45.7 |
| T_4_ | 5 | 10.9 |
| Unknown | 7 | 15.2 |
| Lymph node status |  |  |
| Negative | 22 | 47.8 |
| Positive | 12 | 26.1 |
| Unknown | 12 | 26.1 |
| Distant metastasis |  |  |
| M_0_ | 21 | 45.7 |
| Unknown | 25 | 54.3 |
| Type of surgery |  |  |
| RNU | 21 | 46.7 |
| RN | 13 | 28.3 |
| Ureterectomy | 5 | 10.9 |
| Other types | 2 | 4.3 |
| Unknown | 5 | 10.9 |
| Lymphadenectomy |  |  |
| Yes | 5 | 10.9 |
| No/unknown | 41 | 89.1 |
| Chemotherapy |  |  |
| Yes | 11 | 23.9 |
| No/unknown | 35 | 76.1 |
| Radiation |  |  |
| Yes | 4 | 8.7 |
| No/unknown | 42 | 91.3 |

**Table S13.** Univariate regression analysis of pathologic classification associated with overall survival of LELC of the upper urinary tract patients. LELC, lymphoepithelioma-like carcinoma, HR, hazard ratio; CI, confidence interval. The bold values were applied to highlight *P*-values which had statistically significance (i.e. *P* < 0.05).

| **Pathologic classification** | **HR** | **95% CI** | ***P* value** |
| --- | --- | --- | --- |
| Pure subtype | 1.000 | - | - |
| Focal subtype | 34.638 | 3.708-323.562 | **0.002** |
| Predominant subtype | 8.627 | 0.999-74.500 | 0.050 |
| Mixed subtype | 2.780 | 0.246-31.427 | 0.409 |

**Supplementary figures**

**
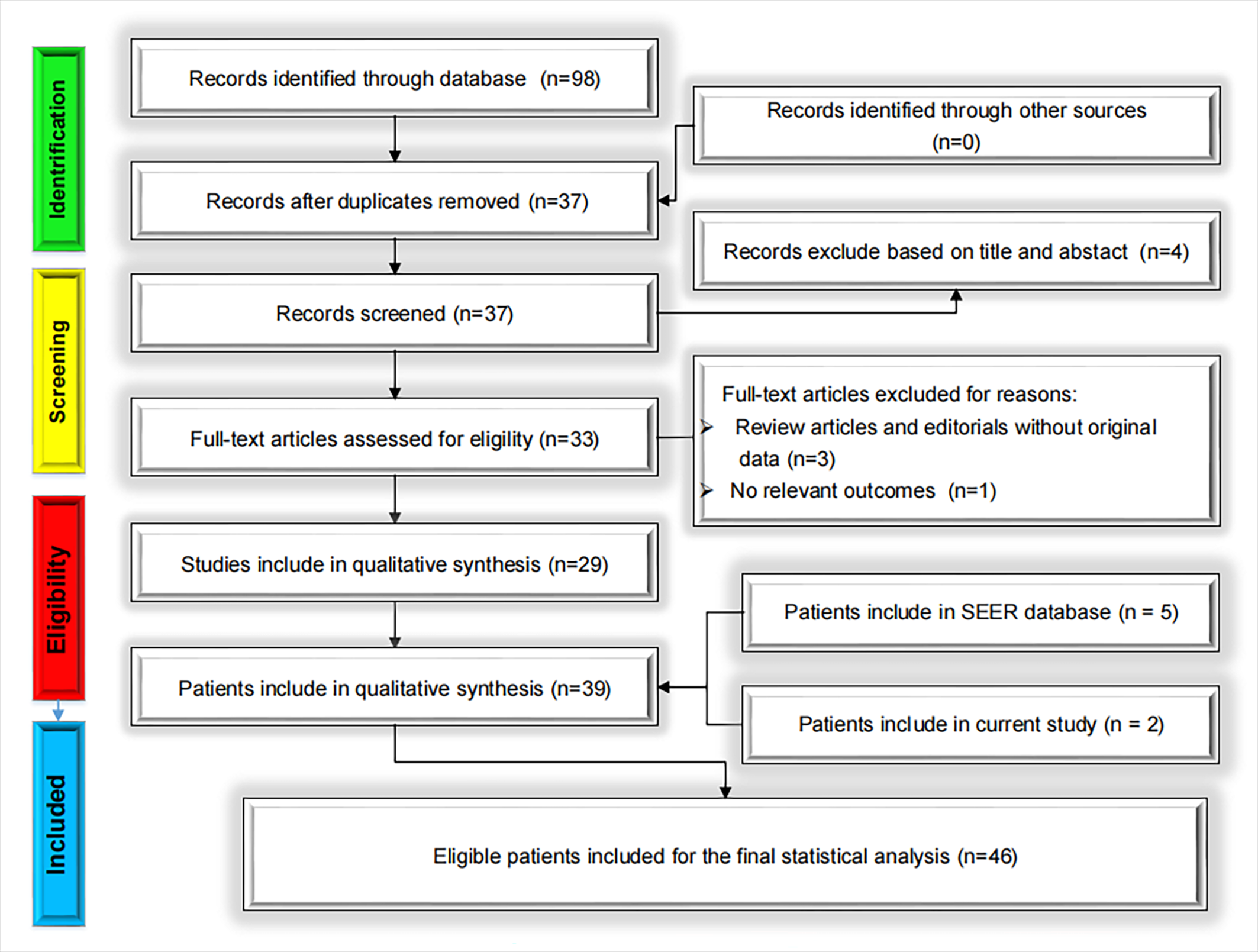
**

**Fig. S1.** Workflow diagram of the selection process for patients with lymphoepithelioma-like carcinoma of the upper urinary tract.


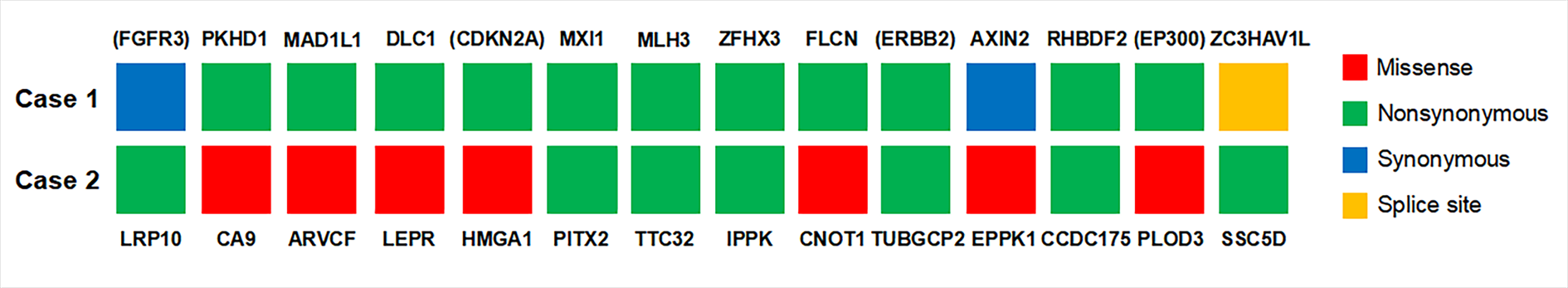


**Fig. S2.** Somatic mutation heatmap of case 1 and 2. Red, green, blue, yellow squares represent missense, nonsynonymous, synonymous, and splice site mutations, respectively. The genes with parentheses were mutation hotspots founded in the Cancer Hotspots website (http://www.cancerhotspots.org/). All the experiments were repeated thrice independently.


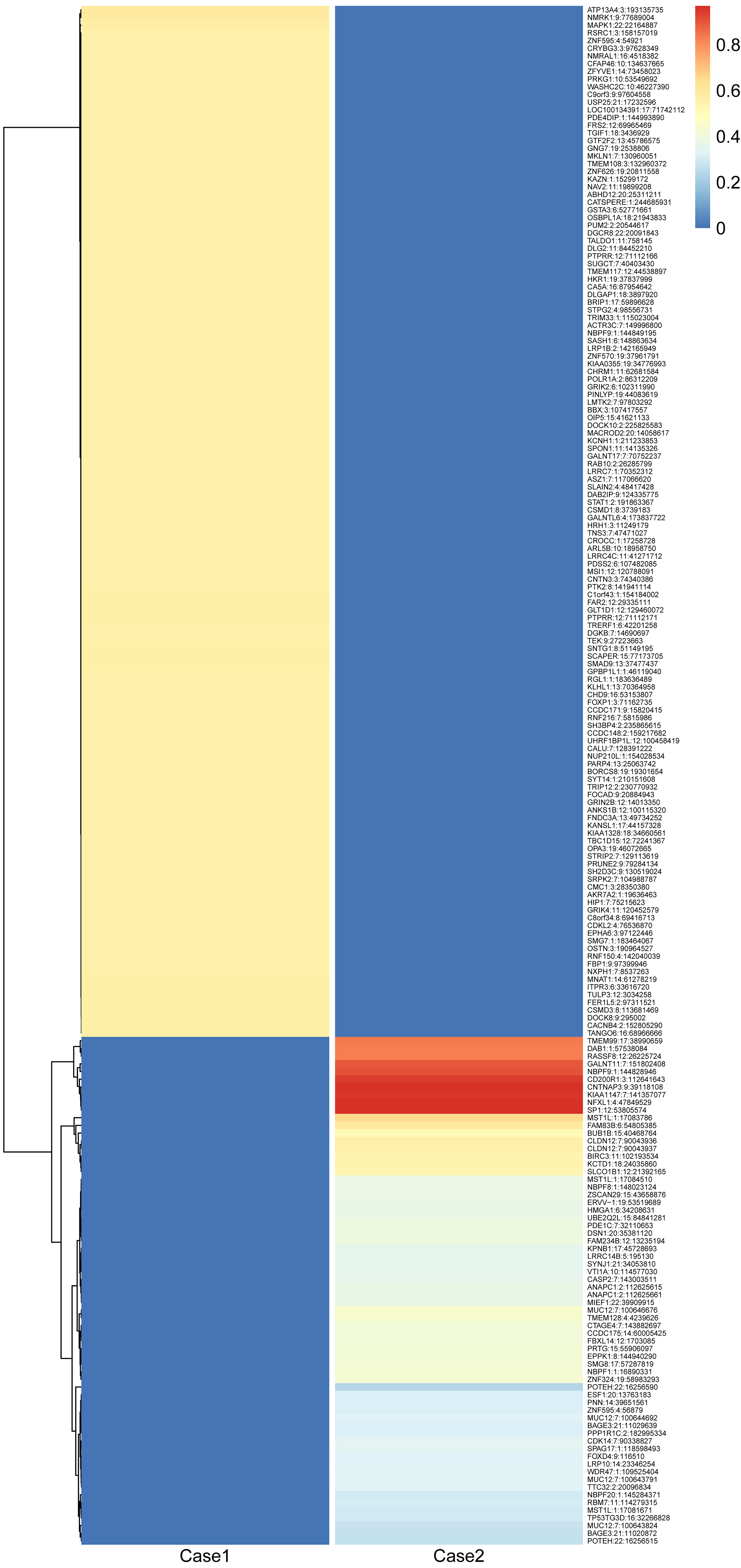


**Fig. S3.** Graph of the results of mutated cancer cell frequency. The horizontal coordinate indicates case numbers (*n* = 2), and the vertical coordinate is the gene in which the mutated locus is located. The mutated cell frequency can be represented by various of colors. From blue to red, the mutated cell frequencies are risen up, and the range is from 0 to 1. All the experiments were repeated thrice independently.
